# Spherical Ni_3_S_2_/Fe‐NiP*_x_* Magic Cube with Ultrahigh Water/Seawater Oxidation Efficiency

**DOI:** 10.1002/advs.202104846

**Published:** 2022-01-12

**Authors:** Xu Luo, Pengxia Ji, Pengyan Wang, Xin Tan, Lei Chen, Shichun Mu

**Affiliations:** ^1^ State Key Laboratory of Advanced Technology for Materials Synthesis and Processing Wuhan University of Technology Wuhan 430070 China; ^2^ Foshan Xianhu Laboratory Foshan 528200 China; ^3^ State Key Laboratory of Silicate Materials for Architectures Wuhan University of Technology Wuhan 430070 China

**Keywords:** double anion heterostructure, oxygen evolution reaction, reconstruction, seawater oxidation, spherical magic cube

## Abstract

The rational construction of earth‐abundant and advanced electrocatalysts for oxygen evolution reaction (OER) is extremely desired and significant to seawater electrolysis. Herein, by directly etching Ni_3_S_2_ nanosheets through potassium ferricyanide, a novel self‐sacrificing template strategy is proposed to realize the in situ growth of NiFe‐based Prussian blue analogs (NiFe PBA) on Ni_3_S_2_ in an interfacial redox reaction. The well‐designed Ni_3_S_2_@NiFe PBA composite as precursor displays a unique spherical magic cube architecture composed of nanocubes, which even maintains after a phosphating treatment to obtain the derived Ni_3_S_2_/Fe‐NiP*_x_* on nickel foam. Specifically, in alkaline seawater, the Ni_3_S_2_/Fe‐NiP*_x_* as OER precatalyst marvelously realizes the ultralow overpotentials of 336 and 351 mV at large current densities of 500 and 1000 mA cm^–2^, respectively, with remarkable durability for over 225 h, outperforming most reported advanced OER electrocatalysts. Experimentally, a series of characterization results confirm the reconstruction behavior in the Ni_3_S_2_/Fe‐NiP*_x_* surface, leading to the in situ formation of Ni(OH)_2_/Ni(Fe)OOH with abundant oxygen vacancies and grain boundaries, which constructs the Ni_3_S_2_/Fe‐NiP*_x_* reconstruction system responsible for the remarkable OER catalytic activity. Theoretical calculation results further verify the enhanced OER activity for Ni_3_S_2_/Fe‐NiP*_x_* reconstruction system, and unveil that the Fe‐Ni_2_P/FeOOH as active origin contributes to the central OER activity.

## Graphical abstract

Well‐designed spherical Ni_3_S_2_/Fe‐NiP*_x_* magic cube architecture stacked by nanocubes is readily reconstructed into Ni(OH)_2_/Ni(Fe)OOH with abundant active sites, cocontributing to ultrahigh‐efficiency seawater oxidation.

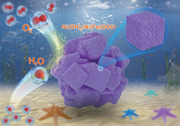

## 1 Introduction

Hydrogen, as a sustainable clean energy, is critical to address the urgent environmental crisis and insufficient fossil energy supply. Among various methods, the hydrogen production by electrochemical water splitting gradually become the mainstream.^[^ ^1^, ^2^, ^3^ ^]^ In general, the water splitting is composed of two half‐reactions, namely the anodic oxygen evolution reaction (OER) and the cathodic hydrogen evolution reaction (HER). Especially, the OER involves multiple proton‐coupled electron transfer, and therefore suffers from a more sluggish dynamics than HER, which usually requires a high overpotential, significantly restricting the efficiency of electrocatalytic water splitting.^[^ ^4^, ^5^ ^]^ However, limited by scarcity and high cost, noble metal catalysts (IrO_2_ and RuO_2_) endowed with high OER catalytic activity are difficult to be applied on a large scale, which stimulates the design and synthesis of nonprecious metal catalysts with high catalytic activity toward overall water splitting and reduce the energy consumption of the reaction.^[^ ^6^, ^7^ ^]^

On the other hand, the technically and economically challenged seawater electrolysis has stricter requirements on the activity and robustness of OER electrocatalysts. Specially, the existence of chloride anions (Cl^–^, ≈0.5 m) in natural seawater would trigger the chlorine evolution reaction (ClER) at the anode and compete with OER.^[^ ^8^ ^]^ Although OER occupies a thermodynamic advantage, its complex four‐electron transfer reaction suffers from a more sluggish kinetics than ClER involving only two electrons.^[^ ^9^ ^]^ Under alkaline conditions (pH > 7.5), Cl^–^ would further react with OH^−^ to form hypochlorite (Cl^−^ + 2OH^−^ → ClO^−^ +H_2_O + 2e^−^), which holds the maximum thermodynamic potential difference (≈480 mV) with OER and results in a convenient potential window for selective OER.^[^ ^10^ ^]^ In summary, alkaline conditions facilitate the selective seawater oxidation, and OER catalysts need to achieve high current density output under 480 mV overpotential to avoid the formation of hypochlorite.^[^ ^11^, ^12^ ^]^ Moreover, chloride corrosion and the poisoning of insoluble precipitations or microbes would also damage the stability and service life of the electrode material, which puts forward higher requirements for the structural stability and corrosion resistance of electrode materials. Considering the abundant natural resources of seawater, which accounts for 96.5% of the global water, and the tremendous advantages of combining seawater splitting technology with other marine‐related power‐generation technologies, the advanced electrochemical seawater splitting is undoubtedly of great significance.^[^ ^9^, ^13^, ^14^ ^]^ Meanwhile, to meet industrial‐grade applications, the development of OER electrocatalysts that can drive large current densities (500–1000 mA cm^–2^) at ultralow potentials and maintain satisfactory stability is also a current research hotspot.^[^ ^15^, ^16^ ^]^

Prussian blue analogues (PBAs), as representative coordination polymer materials, are mainly composed of metal ions centers and cyanide ligand with a general formula of A*_x_* B[M(CN)_6_]·*n*H_2_O (A: alkali metal, Li, Na, K; B and M: transition metal, Ni, Fe, Co, Zn, Mn, etc).^[^ ^17^, ^18^, ^19^ ^]^ The diversity of transition metal ions enables PBAs to possess adjustable metal active sites, accompanied by their inherent characteristic open‐framework structures, and their derived materials (alloys,^[^ ^20^ ^]^ transition metal oxides,^[^ ^21^ ^]^ chalcogenides,^[^ ^22^, ^23^ ^]^ phosphides,^[24]^ etc.) exhibit uniform active sites and large specific surface areas, which have also been widely applied to electrocatalytic water splitting. At present, the reported synthesis strategies of PBAs mainly include co‐precipitation and etching, particularly, the synthesis of PBAs precursors directly through K_3_[M(CN)_6_] (M = Ni, Co, Fe) etching of metals, metal oxides, metal hydroxides, or metal carbonate hydroxides, etc. has been widely investigated.^[^ ^17^ ^]^ Zhang et al. demonstrated an in situ interfacial scaffolding strategy that employed cobalt hydroxide or oxide nanoarrays as the precursor and template for subsequent growth of CoFe PBA nanocubes, and the derived CoFe nitride exhibits excellent electrochemical activity and durability for OER and HER.^[^ ^25^ ^]^ Moreover, they also prepared a variety of PBAs by directly immersing various metal substrates (copper foam, nickel foam, Fe plate, Sn plate, Zn plate, etc.) into K_3_Fe(CN)_6_, and the as‐obtained CuFe oxide derivative after thermal treatment exhibits remarkable OER activity and impressive durability.^[^ ^26^ ^]^ However, most of the reported PBA‐derived nanomaterials focus on homogenous single‐phase structures or single‐anionic bimetallic heterostructures, which partially limits their electrocatalytic performance and electrical conductivity.^[^ ^27^ ^]^ The design of the double anionic heterostructure can compatibly integrate the advantages of differentiated components, which allows precise control of the catalytic activity and other physiochemical properties of the material.

The synergistic effect between S and P anions on the optimization of the catalytic activity of electrode materials can be established based on theoretical calculation,^[^ ^28^ ^]^ which further inspires the rational design of sulfide/phosphide heterostructure accompanied by the introduction of PBAs functional materials. In consideration of the unique metallic property and variable morphology advantages of Ni_3_S_2_ materials with continuous Ni―Ni bonds,^[^ ^29^ ^]^ herein, for the first time we directly employ Ni_3_S_2_ nanosheets as part of the self‐sacrificing template to design and synthesize the Ni_3_S_2_@NiFePBA spherical Rubik's cube architecture in an interfacial redox reaction. After the subsequent thermal phosphating treatment on foam nickel (NF), the well‐designed double anion heterostructure (Ni_3_S_2_/Fe‐NiP*_x_*), with a special spherical magic cube architecture stacked by nanocubes is obtained, presents a multidimensional reaction contact area, which effectively exposes more active sites. During the OER process, the leaching of surface P and partial S elements in Ni_3_S_2_/Fe‐NiP*_x_*/NF precatalysts promotes the rapid surface reconstruction, and results in a defect‐rich Ni(OH)_2_/Ni(Fe)OOH active layer composed of tiny nanoparticles, which effectively inhibits the severe leaching of S elements and modulates the adsorption free energy of intermediates for enhanced OER activity. Detailed electrochemical analysis further reveals splendid OER electrocatalytic activity of Ni_3_S_2_/Fe‐NiP*_x_*/NF as precatalyst. When driving 1000 mA cm^–2^‐ level‐current density, the required overpotential in 1 m KOH electrolytes and alkaline seawater is only 291 and 351 mV, respectively, accompanied by long‐term stability.

## 2 Results and Discussion

### 2.1 Synthesis and Structural Characterizations of Ni_3_S_2_@Fe‐NiP*_x_*/NF

**Scheme 1:**
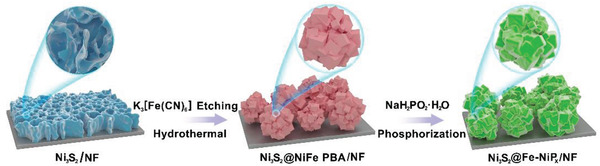
Synthetic illustration of spherical Ni_3_S_2_/Fe‐NiP*_x_* magic cube architecture.

As illustrated in **Scheme** **1**, the Ni_3_S_2_@Fe‐NiP*_x_* spherical Rubik's cube architecture stacked by nanocubes was constructed through a novel self‐sacrificing template strategy and partial phosphating engineering. First, Ni_3_S_2_ nanosheets were synthesized on 3D NF substrate (Ni_3_S_2_/NF) through a simple hydrothermal process. Then, Ni_3_S_2_/NF served as a self‐sacrificial template was directly chemical‐etched with K_3_[Fe(CN)_6_] through a secondary hydrothermal treatment. The interfacial redox reaction and co‐precipitation process induced the in situ formation of NiFe PBA on the surface of Ni_3_S_2_ nanosheets. Then the Ni_3_S_2_@NiFePBA spherical Rubik's cube was established. The subsequent partial phosphating treatment of the Ni_3_S_2_@NiFePBA precursor resulted in a well‐designed Ni_3_S_2_/Fe‐NiP*_x_* heterostructure on NF (Ni_3_S_2_/Fe‐NiP*_x_*/NF) with excellent intrinsic catalytic activity and morphological advantages.

**Figure 1:**
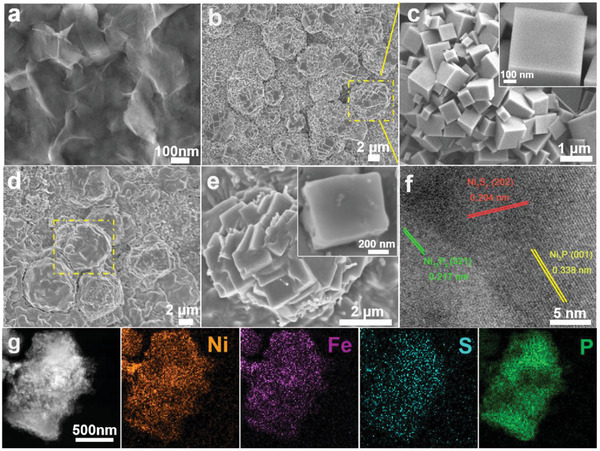
a) FESEM images of Ni_3_S_2_/NF, b,c) Ni_3_S_2_@NiFe PBA, d,e) Ni_3_S_2_/Fe‐NiP*_x_*/NF at different magnifications. f) High‐resolution TEM images of Ni_3_S_2_/Fe‐NiP*_x_*/NF. g) STEM image and the corresponding element mappings of Ni_3_S_2_/Fe‐NiP*_x_*/NF.

In Figures 1a, the Field‐emission scanning electron microscopy (FESEM) image of Ni_3_S_2_ shows an interconnected nanosheet structure that uniformly distributes on the nickel foam (NF) skeleton. After the interface redox reaction with K_3_[Fe(CN)_6_], the PBA nanocubes formed in situ with a size of 400–600 nm are stacked on each other to build a spherical Rubik's cube architecture consisting of nanocubes, which reveals the successful fabrication of NiFePBA with Ni_3_S_2_ nanosheets directly served as a partial sacrificial template (**Figure** **1**b,c). In order to investigate the formation mechanism of the spherical Rubik's cube architecture, NF/NiFe PBA was synthesized by using NF as a precursor template (Figure S1a, Supporting Information). FESEM images in Figure S2a,b (Supporting Information) mainly display uniform distribution of nanocubes, no stacked spherical structures are detected for NF/NiFe PBA. To eliminate the influence of the nanosheet precursor on the synthesis of the spherical Rubik's cube architecture, Ni(OH)_2_ nanosheets were also synthesized as the precursor template to grow NiFe PBA (Figure S1b, Supporting Information). As shown in Figure S2c,d (Supporting Information), the formation of spherical structures is not observed in Ni(OH)_2_@NiFePBA, further confirming the unique morphological advantages of Ni_3_S_2_ nanosheets. As shown in Figure S3 (Supporting Information), FESEM images of Ni_3_S_2_@NiFePBA with different K_3_[Fe(CN)_6_] etching time further explain the morphological evolution of the spherical Rubik's cube architecture. When etched with K_3_[Fe(CN)_6_] for 12 h, the original nanosheet of Ni_3_S_2_ disappears and merges into a spherical structure without complete nanocube, indicating the key role of Ni_3_S_2_ for forming the spherical Rubik's cube, while the reaction time is too short to generate PBA materials. The formation of the spherical Rubik's cube architecture can be detected when the reaction time is extended to 24 h. When further extended to 36 h, the obtained morphology has no obvious change, showing a larger spherical structure size generally.

After phosphating, the obtained Ni_3_S_2_/Fe‐NiP*_x_* on NF basically maintains the initial spherical Rubik's cube structure (Figure 1d), while the corners of the nanocubes are passivated, and the surface becomes rough with some small particles attached (Figure 1e). The high‐resolution TEM (HRTEM) analysis determines the fringe spacings of 0.204, 0.217, and 0.338 nm, corresponding to the (202), (321), and (001) planes of Ni_3_S_2_, Ni_12_P_5_, and Ni_2_P, respectively (Figure 1f). Additionally, the uniform distribution of Ni, Fe, S, and P elements in a single Ni_3_S_2_/Fe‐NiP*_x_* nanocube is further confirmed by energy dispersive X‐ray spectroscopy (EDX) elemental mapping image in Figure 1g.

**Figure 2:**
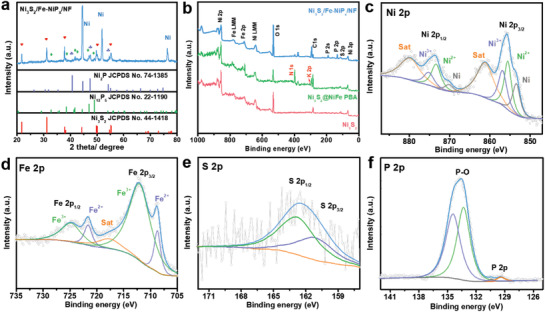
a) XRD patterns of Ni_3_S_2_/Fe‐NiP*_x_*/NF. b) XPS survey pattern of obtained samples. The high‐resolution XPS of c) Ni 2p, d) Fe 2p, e) S 2p, and f) P 2p for Ni_3_S_2_/Fe‐NiP*_x_*/NF.

The powder X‐ray diffraction (XRD) was performed to probe the phase structures of the samples. In the XRD pattern of Ni_3_S_2_/NF (Figure S4a, Supporting Information), the peaks centering at 44.5°, 51.8°, and 76.4° can be indexed to the NF substrate (JCPDS No. 04‐0850), the as‐prepared Ni_3_S_2_ nanosheets are consistent with the standard JCPDS card of No. 44‐1418. After etching with K_3_[Fe(CN)_6_], the additional diffraction peaks at 17.6°, 25.0°, 35.8°, and 54.5° are attributed to the (200), (220), (400), and (600) lattice planes of K_2_FeNi(CN)_6_ (JCPDS No. 20‐0915), manifesting the partial transformation of Ni_3_S_2_ nanosheets to K_2_FeNi(CN)_6_ (Figure S4b, Supporting Information). Simultaneously, the typical cyano‐bridge (C≡N) characteristic was also detected in the Fourier Transform infrared spectroscopy (FTIR) spectrum (Figure S5, Supporting Information).^[30,31]^ After phosphating, the characteristic peaks of NiFe PBA disappear completely and all diffraction peaks are well indexed to Ni_3_S_2_ (JCPDS No.44‐1418), Ni_12_P_5_ (JCPDS No. 22‐1190), and Ni_2_P (JCPDS No. 74‐1385), respectively, except for the unavoidably scraped NF (**Figure** **2**a). This indicates that NiFe PBA/NF mainly formed Fe‐doped NiP*_x_* after phosphating and the derived catalyst coupled with Ni_3_S_2_ to construct a double anion heterostructure, which can be further confirmed by the XRD pattern of Fe‐NiP*_x_*/NF obtained by phosphating NF@NiFe PBA (Figure S6, Supporting Information).

The in situ synthesis process of NiFe PBA and the surface composition of Ni_3_S_2_/Fe‐NiP*_x_*/NF were further analyzed by X‐ray photoelectron spectroscopy (XPS). The appearance of N and K elements in the XPS spectrum of Ni_3_S_2_@NiFePBA/NF (Figure 2b and Figure S7a, Supporting Information) further confirms the successful synthesis of PBA materials with Ni_3_S_2_/NF nanosheets served as a self‐sacrificial template.^[^ ^26^, ^30^ ^]^ In the high‐resolution XPS spectrum of Ni_3_S_2_@NiFePBA/NF (Figure S7b,c), the Ni 2p signal displays a significant positive shift relative to Ni_3_S_2_, and the Fe 2p spectrum mainly shows Fe^2+^ signal instead of Fe^3+^ derived from the K_3_[Fe(CN)_6_] raw material, which strongly proves the interface redox reaction between K_3_[Fe(CN)_6_] and Ni_3_S_2_ during the etching process.^[^ ^11^, ^26^ ^]^

After the phosphating treatment, the coexistence of Ni, Fe, S, and P elements can be confirmed by the XPS survey pattern of Ni_3_S_2_/Fe‐NiP*_x_*/NF (Figure 2b). Besides, the additional O, N, and K elements indicate the inevitable surface oxidation and trace ligand residues, respectively. In the Ni 2p spectrum of Ni_3_S_2_/Fe‐NiP*_x_*/NF (Figure 2c), the two peaks at 855.4 and 873.2 eV are attributed to Ni^2+^ 2p3/2 and Ni^2+^ 2p1/2, while the two peaks at 856.7 and 875.0 eV are ascribed to Ni^3+^ 2p3/2 and Ni^3+^ 2p1/2, indicating the coexistence of Ni^2+^ and Ni^3+^ in the Ni_3_S_2_/Fe‐NiP*_x_* heterostructure.^[^ ^32^, ^33^ ^]^ In addition, the signals located at 861.0 and 879.7 eV are assigned to satellite peaks (labeled “Sat”), and the other two peaks at 853.3 and 870.0 eV are derived from the Ni^0^ in the NF substrate.^[^ ^34^ ^]^ For Fe 2p XPS spectrum (Figure 2d), the binding energy at 708.7 and 721.6 eV can be assigned to Fe 2p3/2 and Fe 2p1/2 components of Fe^2+^, and the higher binding energy located at 712 and 724.8 eV correspond to Fe 2p3/2 and Fe 2p1/2 components of Fe^3+^, with a satellite peak at 717.6 eV.^[^ ^26^, ^35^ ^]^ In Figure 2e, the S 2p spectra of Ni_3_S_2_/Fe‐NiP*_x_*/NF mainly display two signal peaks of S 2p3/2 and S 2p1/2, which are located at the binding energy of 161.1 and 162.8 eV, respectively.^[^ ^36^, ^37^ ^]^ As for the P 2p spectra in Figure 2f, the two peaks at 129.4 and 130.4 eV are ascribed to the P 2p3/2 and P 2p1/2 region of Ni─P bond, and the other two signal peaks at 133.3 and 134.4 eV are attributed to P─O bonds.^[^ ^14^, ^38^ ^]^

The above analysis fully evidences the successful synthesis of Ni_3_S_2_@NiFePBA spherical Rubik's cubes in an interface redox reaction, and the successful transformation into the well‐designed Ni_3_S_2_/Fe‐NiP*_x_* double anion heterostructure without morphological collapse during the low‐temperature phosphating process.

### 2.2 OER Performance of Ni_3_S_2_@Fe‐NiP*_x_*/NF Precatalyst in Alkaline Media

**Figure 3:**
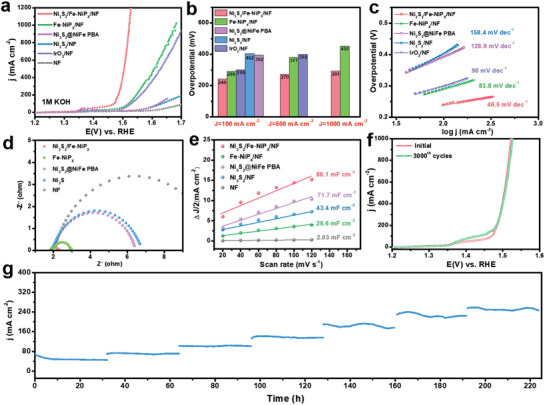
a) OER Polarization curves in 1 m KOH and b) corresponding overpotentials at 100, 500, and 1000 mA cm^–2^ and c) corresponding Tafel plots of the obtained sample. d) EIS Nyquist plots of the Bare NF, Ni_3_S_2_/NF, Ni_3_S_2_@NiFePBA/NF, Fe‐NiP*_x_*/NF, and Ni_3_S_2_/Fe‐NiP*_x_*/NF with a frequency range of 0.01 Hz to 100 kHz. e) Estimated *C*_dl_ values. f) LSV curves before and after 3000 CV cycles of Ni_3_S_2_/Fe‐NiP*_x_*/NF. g) Chronoamperometry curves (at 0.58, 0.6, 0.62, 0.64, 0.66, 0.68, and 0.70 V vs Hg/HgO) of Ni_3_S_2_/Fe‐NiP*_x_*/NF in 1 m KOH.

The electrocatalytic OER performance of Ni_3_S_2_/Fe‐NiP*_x_*/NF, pure Ni_3_S_2_/NF, Ni_3_S_2_@NiFe PBA/NF, and Fe‐NiP*_x_*/NF precatalysts was first evaluated in 1 m KOH electrolytes with a scan rate of 2 mV s^–1^. As benchmark, the IrO_2_ catalyst with controlled mass loading on Ni foam was also evaluated for comparison. After cyclic voltammetry (CV) activation, the obtained polarization curves with iR‐compensated in **Figure** **3**a indicate that the Ni_3_S_2_/Fe‐NiP*_x_*/NF catalyst possesses a remarkable OER electrocatalytic activity compared to other samples. It only requires an ultralow OER overpotential of 240 mV to drive a current density of 100 mA cm^–2^, which is 162, 152, 49, and 58 mV lower than that of Ni_3_S_2_/NF, Ni_3_S_2_@NiFe PBA/NF, Fe‐NiP*_x_*/NF, and IrO_2_/NF, respectively (Figure 3b). In particular, the Ni_3_S_2_/Fe‐NiP*_x_*/NF catalyst even drives larger current densities of 500 and 1000 mA cm^–2^ at the competitive overpotential of 270 and 291 mV, respectively, much lower than that of IrO_2_/NF (398 mV@500 mA cm^–2^) and Fe‐NiP*_x_*/NF (377 mV@500 mA cm^–2^, 450 mV@1000 mA cm^–2^). This manifests a significant leap in OER catalytic performance for synthesizing nickel‐based sulfur phosphide heterostructures by introducing the functional materials PBAs with Ni_3_S_2_ as the sacrificial template.

Figure S8 (Supporting Information) shows the OER polarization curves of Ni_3_S_2_/Fe‐NiP*_x_*/NF with different etching time (12, 24, 36 h) by K_3_[Fe(CN)_6_]. Obviously, Ni_3_S_2_/Fe‐NiP*_x_*/NF with an etching time of 24 h holds the highest OER electrocatalytic activity. Furthermore, Ni_3_S_2_/Fe‐NiP*_x_*/NF also displays a considerably smaller Tafel slope of 46.5 mV dec^–1^ compared to the corresponding values of 83.8, 90, 128.9, and 158.4 mV dec^–1^ for Fe‐NiP*_x_*/NF, IrO_2_/NF, Ni_3_S_2_@NiFe PBA/NF and Ni_3_S_2_/NF, indicating its favorable catalytic reaction kinetics for water oxidation (Figure 3c). Electrochemical impedance spectroscopy (EIS) Nyquist plots (Figure 3d) fitted by the equivalent circuit model of Figure S9 (Supporting Information) reveals the smallest charge transfer resistance (*R*_ct_) for the Ni_3_S_2_/Fe‐NiP*_x_*/NF catalyst, proving that it is equipped with the fastest charge transfer rate in the OER process.

In addition, the electrochemically active surface area (ECSA) of the catalysts was further estimated according to the proportional double‐layer capacitance (*C*_dl_) value. The calculated *C*_dl_ values of Ni_3_S_2_/Fe‐NiP*_x_*/NF, Fe‐NiP*_x_*/NF, Ni_3_S_2_@NiFePBA/NF and Ni_3_S_2_/NF catalysts are 88.1, 26.6, 71.7, and 43.4 mF cm^–2^, respectively (Figure 3e and Figure S10, Supporting Information). The lower *C*_dl_ value of the Fe‐NiP*_x_*/NF catalyst than Ni_3_S_2_/NF indicates that the Fe‐NiP*_x_* nanocube has a smaller electrochemically active surface area than the Ni_3_S_2_/NF nanosheet. This indicates that its excellent OER performance is mainly attributed to its remarkable intrinsic electrocatalytic activity, which also fully confirms the morphological advantages of Ni_3_S_2_/NF nanosheets. The Ni_3_S_2_/Fe‐NiP*_x_*/NF spherical Rubik's cube possesses the largest C_dl_ (88.1 mF cm^–2^), implying that a larger electrochemically active area was constructed by growing NiFe PBA in situ on Ni_3_S_2_ nanosheets and phosphating engineering. To further explore the intrinsic catalytic activity of the catalysts, the current density was normalized by ECSA, the Ni_3_S_2_/Fe‐NiP*_x_*/NF spherical Rubik's cube still displays the optimal intrinsic electrocatalytic activity for OER (Figure S11, Supporting Information), demonstrating that other factors besides ECSA are dedicated to the enhanced OER activity. Furthermore, Ni_3_S_2_/Fe‐NiP*_x_*/NF also has the largest number of active sites and the highest TOF per active site (0.63 S^–1^ at an overpotential of 250 mV) among these samples (Figure S12, Supporting Information). The Faradaic efficiency of Ni_3_S_2_/Fe‐NiP*_x_* in 1 m KOH was measured to study the charge transfer efficiency during the OER. The good consistency between the measured and theoretical O_2_ amounts reveals a Faraday efficiency of nearly 100% for OER (Figure S13, Supporting Information). The above analysis results sufficiently illustrate the ingenious design of the spherical Ni_3_S_2_/Fe‐NiP*_x_*/NF Rubik's cube employing Ni_3_S_2_/NF nanosheets as part of the self‐sacrificial template, which can not only maximize the ECSA and OER active sites, but also compatibly synergize the superior intrinsic OER activity of Fe‐NiP*_x_*, thus resulting in its outstanding OER performance.

In addition to catalytic activity, long‐term stability is also an important criterion for evaluating the eligibility of the catalysts in practical applications. The LSV curve of Ni_3_S_2_/Fe‐NiP*_x_*/NF before and after the 3000‐cycles CV scans (Figure 3f) shows a negligible attenuation. The overpotential increases by only 6 mV, when the current density reaches 1000 mA cm^–2^. In addition, the electrochemical stability of the Ni_3_S_2_/Fe‐NiP*_x_*/NF spherical Rubik's cube was further determined according to the chronoamperometry. As presented in Figure 3g, the Ni_3_S_2_/Fe‐NiP*_x_*/NF electrode can maintain a stable output current density for nearly 225 h under gradient constant potential, which fully confirms the excellent OER electrocatalytic durability of the Ni_3_S_2_/Fe‐NiP*_x_*/NF electrode material under alkaline conditions.

### 2.3 OER Performance of Ni_3_S_2_@Fe‐NiP*_x_*/NF Precatalyst in Alkaline Seawater

**Figure 4:**
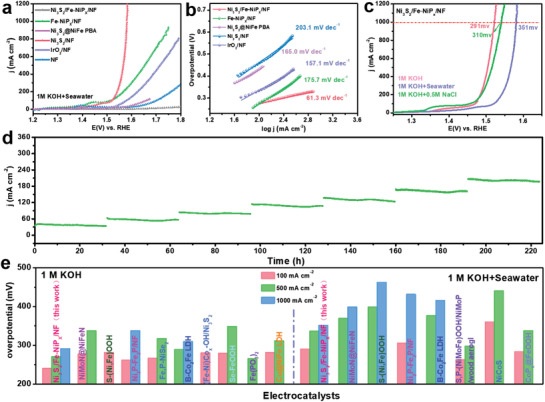
a) OER Polarization curves and b) corresponding Tafel plots of the obtained sample in alkaline natural seawater. c) OER Polarization curves of Ni_3_S_2_/Fe‐NiP*_x_*/NF in different electrolytes. d) Chronoamperometry curves of Ni_3_S_2_/Fe‐NiP*_x_*/NF (at 0.58, 0.6, 0.62, 0.64, 0.66, 0.68, and 0.70 V vs Hg/HgO) in alkaline natural seawater. e) Comparison of the overpotentials required to achieve current densities of 100, 500, and 1000 mA cm^–2^ for OER in 1 m KOH and alkaline natural seawater between Ni_3_S_2_/Fe‐NiP*_x_*/NF and other reported state‐of‐the‐art catalysts.

In consideration of the excellent OER catalytic activity of Ni_3_S_2_/Fe‐NiP*_x_*/NF in 1 m KOH electrolytes, we further evaluated its OER performance in configured alkaline seawater (1 m KOH + seawater) and simulated seawater (1 m KOH + 0.5 m NaCl). The natural seawater was taken from Dalian (Liaoning, China) with a pH of about 7.38, and the alkaline natural seawater prepared with 1 m KOH is a milky white turbid liquid with a pH of about 14.35 (Figure S14, Supporting Information). The measured LSV curve with iR‐compensation is shown in **Figure** **4**a. It can be seen that Ni_3_S_2_/Fe‐NiP*_x_*/NF spherical Rubik's nanocube still presents remarkable OER catalytic activity in alkaline natural seawater electrolyte, only requiring overpotentials of 290 and 336 mV to realize industrial current densities of 100 and 500 mA cm^–2^, respectively. In comparison, the overpotential required for Fe‐NiP*_x_*/NF and IrO_2_/NF with the same load to reach a current density of 500 mA cm^–2^ are 423 and 485 mV, respectively. In particular, at the larger current density of 1000 mA cm^–2^, the overpotential of Ni_3_S_2_/Fe‐NiP*_x_*/NF spherical Rubik's cube is only 351 mV, much lower than the theoretical overpotential value (480 mV) demanded to generate hypochlorite at the anode.

The Tafel slope of the obtained catalysts was also applied to analyze the kinetics of water oxidation in alkaline natural seawater. As shown in Figure 4b, compared with 1 m KOH solutions, the OER kinetics of all catalysts in alkaline natural seawater is significantly slower, the Tafel slopes of Ni_3_S_2_/NF, Ni_3_S_2_@NiFe PBA/NF, Fe‐NiP*_x_*/NF, IrO_2_/NF, and Ni_3_S_2_/Fe‐NiP*_x_*/NF are 203.1, 165.0, 175.7, 157.1 and 61.3 mV dec^–1^, respectively. Among them, the Tafel slope of the Ni_3_S_2_/Fe‐NiP*_x_*/NF (61.3 mV dec^–1^) is much lower than that of the commercial IrO_2_ catalyst and other comparative samples, indicating that the Ni_3_S_2_/Fe‐NiP*_x_*/NF catalyst still possesses a vigorous OER catalytic kinetics in alkaline seawater. The Faradaic efficiency of the Ni_3_S_2_/Fe‐NiP*_x_*/NF electrode in 1 m KOH + seawater was determined to be around 95.7% during the seawater oxidation, manifesting the high selectivity for OER (Figure S15, Supporting Information). When employing Pt/C/NF as the cathode to assemble a two‐electrode system, the Pt/C/NF||Ni_3_S_2_/Fe‐NiP*_x_*/NF pair exhibits remarkable activity for overall water/seawater splitting, substantiating its great potential for practical applications (Figure S16, Supporting Information).

In simulated seawater (1 m KOH + 0.5 m NaCl), the Ni_3_S_2_/Fe‐NiP*_x_*/NF catalyst reveals an attenuated OER activity compared to that in 1 m KOH solutions. The overpotential required to achieve a current density of 1000 mA cm^–2^ is 310 mV, which is merely 19 mV higher than that in 1 m KOH solutions (Figure 4c). The OER catalytic activity of the Ni_3_S_2_/Fe‐NiP*_x_*/NF catalyst in alkaline natural seawater is further reduced, the required overpotential (351 mV) at 1000 mA cm^–2^ is 60 mV higher than in 1 m KOH electrolyte, which may be caused by the impurity ions, bacteria and fine particles in natural seawater, as well as the concealment of surface active sites by insoluble precipitates (Mg(OH)_2_ and Ca(OH)_2_).^[^ ^12^, ^39^ ^]^ Furthermore, the Ni_3_S_2_/Fe‐NiP*_x_*/NF catalyst also displays outstanding OER long‐term stability in alkaline seawater. As shown in Figure 4d, Ni_3_S_2_/Fe‐NiP*_x_*/NF still holds a stable current density output capability under the gradient constant potential of nearly 225 h, without significant attenuation. The corrosion resistance performance of Ni_3_S_2_/Fe‐NiP*_x_* was further evaluated through the corrosion polarization analysis in natural seawater. In Figure S17 (Supporting Information), the Ni_3_S_2_/Fe‐NiP*_x_* catalyst exhibits an optimized corrosion current density compared to Fe‐NiP*_x_*, indicating its higher corrosion resistance after coupling with Ni_3_S_2_. Moreover, the long‐term immersion test in different concentrations of NaCl alkaline electrolytes was implemented to evaluate the corrosion resistance of Ni_3_S_2_/Fe‐NiP*_x_* against high concentrations of Cl^–^. In the optical and SEM images (Figures S18 and S19, Supporting Information), no obvious structural corrosion and collapse were detected in Ni_3_S_2_/Fe‐NiP*_x_* after immersed in 1.5 m NaCl alkaline electrolytes for 20 days, confirming its excellent structural stability and corrosion resistance. Undoubtedly, as a non‐noble metal‐based OER precatalyst, the spherical Ni_3_S_2_/Fe‐NiP*_x_* Rubik's cube also has very strongly competitive performance among most of the latest reported state‐of‐the‐art catalysts, whether in 1 m KOH solutions or alkaline seawater electrolytes. (Figure 4e and Table S1, Supporting Information)

### 2.4 Active Sites for Oxygen Evolution Catalysis

**Figure 5:**
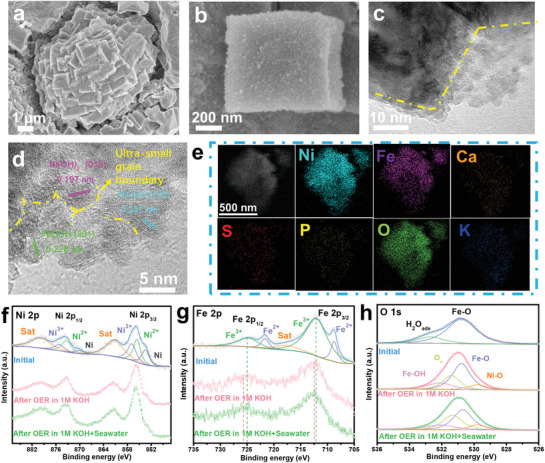
a,b) FESEM images of Ni_3_S_2_/Fe‐NiP*_x_*/NF after OER test in 1 m KOH solution. c,d) High‐resolution TEM images of Ni_3_S_2_/Fe‐NiP*_x_*/NF after OER test in 1 m KOH at different magnifications. e) STEM image and the corresponding element mappings of Ni_3_S_2_/Fe‐NiP*_x_*/NF after OER test in alkaline natural seawater. High‐resolution XPS of f) Ni 2p, g) Fe 2p, and h) O1s of Ni_3_S_2_/Fe‐NiP*_x_*/NF before and after OER test.

For the OER, the formation of metal (oxy)hydroxides on the surface of the catalyst and the leach of the original elements will trigger different degrees of reconstruction in morphology and composition, and the resulting species after the reconstruction is considered to be the true OER active species.^[^ ^40^ ^]^ To further understand the reconstruction phenomena and the real active sites of the Ni_3_S_2_/Fe‐NiP*_x_*/NF catalyst during the OER process, its morphology, composition, and chemical state after OER test were investigated by FESEM, TEM, XPS, XRD, and Raman. To facilitate analysis, the reconstructed Ni_3_S_2_/Fe‐NiP*_x_*/NF is named by Ni_3_S_2_/Fe‐NiP*_x_*/NF‐R. As shown in **Figure** **5**a,b and Figure S20 (Supporting Information), the spherical Rubik's cube of Ni_3_S_2_/Fe‐NiP*_x_*/NF is well maintained with a rougher surface after long‐term OER testing in 1 m KOH and even alkaline seawater, indicating the occurrence of the surface reconstruction process and further proving the excellent structural stability and corrosion resistance of the electrode material. TEM images in Figure S21 (Supporting Information) reveal the appearance of thin layers on the surface of the nanocube, which was further confirmed by the corresponding HRTEM images (Figure 5c). Different from the relatively regular crystal lattice arrangement of the Ni_3_S_2_/Fe‐NiP*_x_*/NF precatalyst (Figure S22, Supporting Information), Ni_3_S_2_/Fe‐NiP*_x_*/NF‐R presents highly disordered lattice fringes, consisting of numerous low‐crystalline nanoparticles, which endow it with abundant grain boundaries. In Figure 5d, the observed the fringe spacings of 0.197, 0.237, 0.228 nm match the (018), (102), and (301) lattice plane of Ni(OH)_2_ (JCPDS No. 38‐0715), NiOOH (JCPDS No. 06‐0075), and FeOOH (JCPDS No. 18‐0639) species, respectively. This undoubtedly determines the formation of the Ni(OH)_2_/Ni(Fe)OOH active material on the surface of Ni_3_S_2_/Fe‐NiP*_x_*/NF during the reconstruction process, which can also be detected for Ni_3_S_2_/Fe‐NiP*_x_*/NF‐R in alkaline seawater (Figure S23, Supporting Information). The EDX elemental mappings reveal the weaker S, P element, and enhanced O element signals for Ni_3_S_2_/Fe‐NiP*_x_*/NF‐R, indicating that the leaching of S and P elements and the intense oxidation process during OER in 1 m KOH and alkaline seawater (Figure 5e and Figure S24, Supporting Information). In particular, the additional Ca element signals derived from seawater can be detected for Ni_3_S_2_/Fe‐NiP*_x_*/NF‐R in alkaline seawater, according to the XPS results (Figure S25a, Supporting Information).

XPS analysis further confirms the loss of S and P elements for Ni_3_S_2_/Fe‐NiP*_x_*/NF‐R (Figure S25b,c, Supporting Information). Among them, the P signals disappear completely, while the weak S element signal can still be detected, revealing the partial leaching of the S element, which can be confirmed by the specific atomic ratio variation of Ni_3_S_2_/Fe‐NiP*_x_*/NF before and after reconstruction (Figure S26a, Supporting Information). The inductively coupled plasma‐optical emission spectrometry (ICP‐OES) test of the electrolyte for Ni_3_S_2_/Fe‐NiP*_x_*/NF after reconstruction manifests that more P elements were leached compared to S (Figure S26b, Supporting Information). This evidences that the in situ generated Ni(OH)_2_/Ni(Fe)OOH during the rapid reconstruction process can further suppress the leaching of elemental S. In the Ni 2p XPS spectra of Ni_3_S_2_/Fe‐NiP*_x_*/NF‐R (Figure 5f), the signal peak originating from the NF substrate is weakened, indicating the surface oxidation of Ni^0^. For Fe 2p XPS spectra (Figure 5g), the two peaks ascribed to Fe^2+^ at 708.7 and 721.6 eV disappear, and Fe^3+^ 2p1/2 and Fe^3+^ 2p3/2 shift to higher binding energy, verifying that the surface Fe ions were oxidized to a higher valence state. From the O 1s XPS spectra (Figure 5h), it confirms the oxidation of Ni and Fe and the formation of oxygen vacancies. Furthermore, the intense electron spin resonance (ESR) signal with g‐factor = 2.003 for Ni_3_S_2_/Fe‐NiP*_x_*/NF‐R (Figure S27, Supporting Information) further verifies the unpaired electrons trapped at O‐vacancies. The O‐vacancies formed may be attributed to the rapid oxidative reconstruction process of the electrode material and play a positive role in the OER activity.^[^ ^41^ ^]^

The XRD pattern (Figure S28, Supporting Information) still shows the characteristic diffraction peaks of Ni_3_S_2_, Ni_12_P_5_, and Ni_2_P, with a relative intensity reduction compared to the initial Ni_3_S_2_/Fe‐NiP*_x_*/NF, which further proves the structural stability of Ni_3_S_2_/Fe‐NiP*_x_*/NF at large current densities, manifesting that the reconstruction occurs only at the surface level.^[^ ^40^ ^]^ Moreover, the Raman spectroscopy in Figure S29 (Supporting Information) further confirms the formation of Ni(Fe)OOH. The peak around 691.7 cm^–1^ corresponds to the Fe–O vibrations in FeOOH, and the other two peaks at 486.8 and 546.5 cm^–1^ can be assigned to the E_g_ bending vibration and the A_1g_ stretching vibration modes of Ni–O in NiOOH.^[^ ^42^, ^43^, ^44^ ^]^ The broadening of the peak position and the blue‐shifted or red‐shifted for Ni‐O mode may be due to the structural defects of NiOOH or the presence of Ni(OH)_2_.^[^ ^43^, ^45^ ^]^

**Figure 6:**
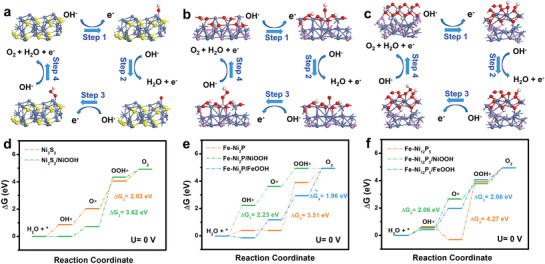
Four‐electron mechanism of OER on a) Ni_3_S_2_; b) Fe‐Ni_2_P/FeOOH; and c) Fe‐Ni_12_P_5_/NiOOH model; d–f) Calculated free‐energy diagram of OER intermediates at zero potential (*U* = 0).

To gain insights into the surface reconstruction and the origin of the intrinsic activity, the energy barriers of the OER intermediates adsorption were analyzed by density functional theory (DFT) calculations. The previous analysis reveals the reconstruction behavior of Ni_3_S_2_/Fe‐NiP*_x_* surface to oxyhydroxides during the OER process. Therefore, the structure models of Ni_3_S_2_, Fe‐Ni_2_P, Fe‐Ni_12_P_5_, and their corresponding hybrids formed after reconstruction (Ni_3_S_2_/NiOOH, Fe‐Ni_2_P/NiOOH, Fe‐Ni_2_P/FeOOH, Fe‐Ni_12_P_5_/NiOOH, Fe‐Ni_12_P_5_/FeOOH) were constructed for DFT calculations in alkaline media. The relevant theoretical models are presented in **Figure** **6**a‐c and Figure S30 (Supporting Information), and the Gibbs free energy diagrams at zero potential (*U* = 0) are summarized in Figure 6d‐f. Theoretically, the OER process involves four proton‐transfer steps in alkaline media, in which the step with the highest Gibbs free energy change (Δ*G*) is determined as the rate determining step (RDS). Based on calculation results, the RDS for Ni_3_S_2_, Fe‐Ni_2_P, Fe‐Ni_12_P_5_ are all the formation of OOH* from O*, with the Δ*G* of 2.03, 3.51, and 4.27 eV, respectively. When coupling Ni_3_S_2_ with NiOOH into the Ni_3_S_2_/NiOOH reconstruction system, the formation of OOH* is still determined as RDS, with an increased energy barrier (3.62 eV), suggesting a more unfavorable OER kinetics. In contrast, the reconstruction system of Fe‐Ni_2_P and Fe‐Ni_12_P_5_ coupled with corresponding oxyhydroxides (NiOOH or FeOOH) can significantly optimize the adsorption free energy of OER intermediates, and the Fe‐Ni_2_P/FeOOH system holds the lowest Δ*G* (1.96 eV) for the rate determining step (the formation of O_2_), demonstrating the major origin of OER intrinsic activity. In brief, DFT calculation results certify the improvement of the OER catalytic activity of the Ni_3_S_2_/Fe‐NiP*_x_* reconstruction system, and the Fe‐Ni_2_P/FeOOH as the active origin, contributing excellent OER activity to the Ni_3_S_2_/Fe‐NiP*_x_* reconstruction system, which is consistent with the results of electrochemical analysis.

According to the above analysis, the Ni_3_S_2_/Fe‐NiP*_x_*/NF catalyst undergoes a rapid oxidative reconstruction process during OER electrocatalysis, and the in situ formed Ni(OH)_2_/Ni(Fe)OOH with abundant oxygen vacancies and grain boundaries evolved from the surface of Ni_3_S_2_/Fe‐NiP*_x_*/NF, accompanying by the leaching of P and partial S elements. The Ni(OH)_2_/Ni(Fe)OOH covering on the surface of the nanocube in spherical suppresses the further leaching of the S element and contributes to excellent OER catalytic activity, of which the Fe‐Ni_2_P/FeOOH system is the central active origin.

## 3 Conclusion

In summary, this work presents a novel self‐sacrificing template strategy to successfully design and build a unique spherical Ni_3_S_2_/Fe‐NiP*_x_*/NF magic cube (Rubik's cube) architecture consisting of nanocubes for seawater oxidation. A series of characterization results reveal the surface reconstruction and elucidate the mechanism. The leaching of surface P and partial S elements triggers the in situ formation of Ni(OH)_2_/Ni(Fe)OOH active species with rich oxygen vacancies and grain boundaries between Ni(OH)_2_, NiOOH, and FeOOH. DFT calculation results confirm that the coupling of Fe‐Ni_2_P and Fe‐Ni_12_P_5_ with the corresponding reconstructed species (FeOOH or NiOOH) can effectively modulate the adsorption free energy of OER active intermediates, resulting in lower Gibbs free energy change. Notably, the Fe‐Ni_2_P/FeOOH hybrid system contributes to the central OER performance as the active origin. Especially, when the current density reaches 1000 mA cm^–2^, it only requires the overpotentials of 291 and 351mV, respectively, in alkaline solutions and alkaline seawater. Simultaneously, it shows outstanding electrochemical stability for seawater oxidation as high as 225 h. Accordingly, we believe that our work provides new ideas for the development of high‐efficiency and low‐cost OER electrocatalysts for seawater splitting, and inspires the rational construction of other functional materials for electrochemical applications.

## Conflict of Interest

The authors declare no conflict of interest.

## Supporting information

Supporting Information

## Data Availability

Research data are not shared.
