# Supplementary material for: Spherical Ni3S2/Fe‐NiP x Magic Cube with Ultrahigh Water/Seawater Oxidation Efficiency
Source: Adv Sci (Weinh). 2022 Jan 12;9(7):2104846. doi: 10.1002/advs.202104846 (PMC8895145; doi:10.1002/advs.202104846)
Supplement: Supplementary file 1 — Supporting Information [file ADVS-9-2104846-s001.pdf]

WILEY-VCH

© 2020 Wiley-VCH GmbH

ADVANCED SCIENCE
Open Access

Supporting Information

for *Adv. Sci.*, DOI: 10.1002/advs.202104846

Spherical Ni_3S_2/Fe-NiP_x Magic Cube with Ultrahigh Water/Seawater Oxidation Efficiency

*Xu Luo, Pengxia Ji, Pengyan Wang, Xin Tan, Lei Chen, and Shichun Mu**

Supporting Information

Spherical Ni_3S_2/Fe-NiP_x Magic Cube with Ultrahigh Water/Seawater Oxidation Efficiency

Xu Luo[1,2], Pengxia Ji[1], Pengyan Wang[1], Xin Tan[3], Lei Chen, Shichun Mu[1,2]*

X. Luo, P. Ji, P. Wang, X. Tan, L. Chen, Prof. S. Mu

[1]State Key Laboratory of Advanced Technology for Materials Synthesis and Processing, Wuhan University of Technology, Wuhan 430070, China

[2]X. Luo, Prof. S. Mu

Foshan Xianhu Laboratory, Foshan 528200, China

[3]State Key Laboratory of Silicate Materials for Architectures, Wuhan University of Technology, Wuhan 430070, China

* Corresponding author

E-mail: msc@whut.edu.cn;

Materials: Nickel nitrate hexahydrate ($Ni(NO_3)_2{\cdot}6H_2O$), thiourea (CH_4N_2S), ammonium fluoride (NH_4F), potassium hexacyanoferrate ($K_3[Fe(CN)_6]$) and Potassium hydroxide (KOH) were purchased from Sinopharm Chemical Reagent Co., Ltd. Hydrochloric acid (HCl) and acetone were purchased from Shanghai Chemical Reagent Company. IrO_2 and Nafion (5 wt %) were purchased from Sigma-Aldrich. All reagents were used directly without purification.

Materials synthesis

Pretreatment of Nickel foam (NF): First, NF (2 cm × 3 cm) was cleaned by sequentially sonicating in acetone and 3M HCl solution for 15 minutes. Then, it was rinsed with deionized (DI) water and ethanol for several times and vacuum dry at 60°C for 12 hours.

Synthesis of Ni_3S_2 nanosheets on NF: Ni_3S_2 nanosheets were synthesized on NF through a simple hydrothermal method. 1 mmol $Ni(NO_3)_2{\cdot}6H_2O$, 0.5 mmol thiourea and 2mmol NH_4F were dissolved in 20 mL DI water. After vigorous stirring for 30 min, the obtained solution was transferred to a 50 mL Teflon-lined stainless-steel autoclave with the pretreated NF, then maintained at 120°C for 8 h. After cooling down room temperature, the Ni_3S_2/NF was taken out and washed with DI water and ethanol, followed by vacuum drying at 60℃ for 12 h.

Synthesis of Ni_3S_2@NiFe PBA: The as-synthesized Ni_3S_2/NF was put in a 50 mL Teflon-lined stainless-steel autoclave containing 20 mL $K_3[Fe(CN)_6]$ solution (0.2 mmol), and the second hydrothermal reaction was carried out at 90°C for 24 h. Subsequently, the sample was carefully rinsed with DI water and ethanol at least 5

times and dried at 60 °C in vacuum oven for 12 h to obtain Ni_3S_2@NiFe PBA/NF composite material. Extend (36 h) or shorten (12 h) the holding time under the same conditions to obtain Ni_3S_2@NiFe PBA/NF-12, Ni_3S_2@NiFe PBA/NF-36.

Synthesis of Ni_3S_2/Fe-NiP_x/NF: Ni_3S_2/Fe-NiP_x/NF was synthesized through a typical phosphating process, the Ni_3S_2/NiFe PBA/NF precursor and 1g $NaH_2PO_2 \cdot H_2O$ powders were placed at the center and upstream side of the tube furnace. Afterward, the furnace was heated to 350 °C with heating rate of 2°C min^{-1} under a high-purity Ar atmosphere, maintained for 2 h. After cooling down to room temperature, the Ni_3S_2@Fe-NiP_x/NF was obtained, and the calculated mass loading was about 18.25 mg/cm^2.

Synthesis of NF@NiFe PBA and Fe-NiP_x/NF: The preparation process of NF@NiFe PBA was similar to that of Ni_3S_2@NiFe PBA, except for replacing the Ni_3S_2 nanosheets with the pretreated nickel foam. The Fe-NiP_x/NF is synthesized with NF@NiFe PBA as the precursor through the same phosphating process.

Preparation of IrO_2 on NF (IrO_2/NF): To prepare the IrO_2 electrode, 50 mg commercial IrO_2 was uniformly dispersed in a mixture solution containing 950 μL water/ethanol (v/v, 1:1) mixture and 50 μL 5 wt % Nafion solution. After continuous sonication for 40 minutes, the homogeneous catalyst ink was dropped onto a nickel foam(working area: 0.5cm×0.5cm) with a controlled load of about 18.25 mg/cm^2, which directly served as working electrode after drying in air overnight.

Characterization:

The morphology and microstructures of as-prepared samples were characterized by

field emission scanning electron microscope (FESEM, Zeiss Ultra Plus) and transmission electron microscope (TEM, JEM2100F). The elemental mapping of the samples was performed by energy-dispersive X-ray spectroscope (EDX) accessory attached to the TEM. The crystalline structures information was obtained by X-ray powder diffraction (XRD, Bruker D8 Advance) with Cu K α radiation (λ = 0.15406 nm).

X-ray photoelectron spectroscopy (XPS) was performed on a VG MultiLab 2000 spectrometer. Fourier transform infrared spectroscopy (FT-IR) was conducted on Nicolet IS5 (Thermo Fisher Scientific Inc.). The Raman spectra of the materials were obtained by LabRAM HR spectrometer (Horiba Jibin Yvon), with argon ion laser (532 nm) as the excitation light source. The inductively-coupled plasma optical emission spectrometry (ICP-OES) was conducted on Teledyne Leeman Labs Prodigy 7. Electron spin resonance (ESR) spectra were obtained from a Bruker A300 spectrometer.

Electrochemical Measurements:

All electrochemical measurements were conducted on a CHI 660E electrochemical workstation in a standard three-electrode system, which was assembled by employing the as-synthesized catalysts (working area, 0.5cm×0.5cm) as the working electrode, Hg/HgO electrode as the reference electrode and graphite rod as the counter electrode. The catalytic activity of the electrode materials was evaluated in 1 M KOH, alkaline seawater (1 M KOH + seawater), simulated seawater (1 M KOH + 0.5 M NaCl),

respectively. All the electrochemical measurements were calibrated to the reversible hydrogen electrode (RHE) by the equation of $E_{RHE} = E_{(Hg/HgO)} + 0.098 + 0.059 \times pH$. The polarization curves in different electrolytes were obtained by using linear sweep voltammetry (LSV) at a scan rate of 2 mV s^{-1}, with 90% iR compensation ($E_{compensated} = E_{measured} - iRs$). Electrochemical impedance spectroscopy (EIS) was performed at the overpotential of 360 mV with the frequency range from 0.01 Hz to 100 kHz, and the charge-transfer resistance (R_{ct}) values were fitted by the equivalent circuit model. For long-term stability measurements, CV methods and chronoamperometry at the gradient constant potential (0.58, 0.6, 0.62, 0.64, 0.66, 0.68 and 0.70 V vs. Hg/HgO) were performed. The electrochemical active surface area (ECSA) of obtained samples was estimated by the double layer capacitance (C_{dl}), which determined by typical cycle-voltage measurements at various scan rates (20, 40, 60, 80, 100, 120 mV s^{-1}) in a non-faradaic region of 0.2-0.3 V (vs. Hg/HgO). To calculate the ECSA, the C_{dl} value of the bare NF was used as a standard instead of the general specific capacitance (C_s) according to the formula:

$$X_{ECSA} = \frac{C_{dl}(catalyst)\ mF\ cm^{-2}}{C_{dl}(NF) mF\ cm^{-2} \cdot per\ ECSA\ cm^{-2}}$$

The Faradaic efficiency (FE) was obtained by comparing the measured O_2 gas amount with the theoretical gas amount according to the formula:

$$\eta_{Faraday} = V_{experimental} / V_{theoretical}$$

where *the* theoretical yield of O_2 ($V_{theoretical}$) can be calculated as:

$$V_{theoretical} = I \cdot t \cdot V_m \,/\, n \cdot F$$

where the I is the measured current in the experiment, t is the measured time, V_m is

the molar volume of O_2 in one mol, n is the number of electrons required for one molecule of O_2 (here n = 4), F is the Faraday's constant (96485, C mol^{-1}).

The number of active sites was quantified by cyclic voltammetry (CV) measurements in phosphate buffered saline solution (PBS, pH=7)[1]. Subsequently, the absolute components of the voltammetric charges (cathodic and anodic) recorded during one single measurement was added. Supposing one electron redox process, the number of active sites (n) of catalysts was calculated by dividing half of the absolute charge by the Faraday constant, which is indicated by the following formula:

$$n = Q/2F = It/2F = IV/2Fv$$

where Q is the voltammetric charge, F is the Faraday constant (96485, C mol^{-1}), I is the current (A), t is the time (s), V is the voltage (V), v is the scanning rate (V s^{-1}).

The TOF (s^{-1}) values were calculated with the following equation:

$$TOF = I/mnF$$

where I is current (A) during the linear sweep voltammetry (LSV) tests in 1 M KOH, n is the number of active sites (mol), F is the Faraday constant (C mol^{-1}), m is the factor (m for hydrogen evolution and oxygen evolution reactions are 2 and 4, respectively).

The overall water/seawater splitting performance without iR compensation was implemented by assembling a two-electrode device with the as-prepared sample as the anode electrode and the HER-active Pt/C as the cathode electrode. The polarization curve was recorded within the potential range of 1 ~ 2.5 V, with a scan rate of 5 mV s^{-1}.

Theoretical calculation Details: Density Functional Theory (DFT) calculations were performed by the CASTEP module of Materials Studio[2, 3], and the Perdew-Burke-Ernzerhof (PBE) functional within the generalized gradient approximation (GGA) was selected to describe the electron exchange-correlation[4]. The projected augmented wave (PAW) method and plane-wave basis set were utilized with energy cutoffs of 300 eV. All cell structures were optimized until the forces on each atom were below 0.05 eV/Å with the iterative convergence of energy of 10^{-5} eV.

The oxygen evolution reaction in alkaline medium involves four proton-transfer steps:

$$* + OH^- \rightarrow OH* + e^- \quad (1)$$

$$OH* + OH^- \rightarrow O* + H_2O + e^- \quad (2)$$

$$O* + OH^- \rightarrow OOH* + e^- \quad (3)$$

$$OOH* + OH^- \rightarrow O_2 + H_2O + e^- \quad (4)$$

The Gibbs free energy of the adsorption of oxygen-containing intermediates (OH*, O* and OOH*) can be obtained by the equation:

$$\Delta G = \Delta E + \Delta E_{ZPE} - T\Delta S$$

where the ΔE is the reaction energy calculated directly using DFT, ΔE_{ZPE} is the change in zero-point energy and ΔS is the entropy change. Here the $\Delta ZPE - T\Delta S$ are 0.06, 0.37 and 0.44 eV for O*, OH* and OOH*, respectively, as proposed by Li et al [5].

According to the above equations and the calculated adsorption free energy of oxygen-containing intermediates M* (ΔG_{M*}), when the applied electrode potential (U)

is 0 V, the free energy differences for each step (ΔG_i, i=1,4) during the OER process can be calculated by the following equation[6]:

$$\Delta G_1 = \Delta G_{OH*} \quad (5)$$

$$\Delta G_2 = \Delta G_{O*} - \Delta G_{OH*} \quad (6)$$

$$\Delta G_3 = \Delta G_{OOH*} - \Delta G_{O*} \quad (7)$$

$$\Delta G_4 = 4.92[eV] - \Delta G_{OOH*} \quad (8)$$

The theoretical OER overpotential of the catalyst is determined by the potential-determining step, which is equal to η_{OER} = max [ΔG_1, ΔG_2, ΔG_3, ΔG_4]/e - 1.23[V]

Supplementary Figures

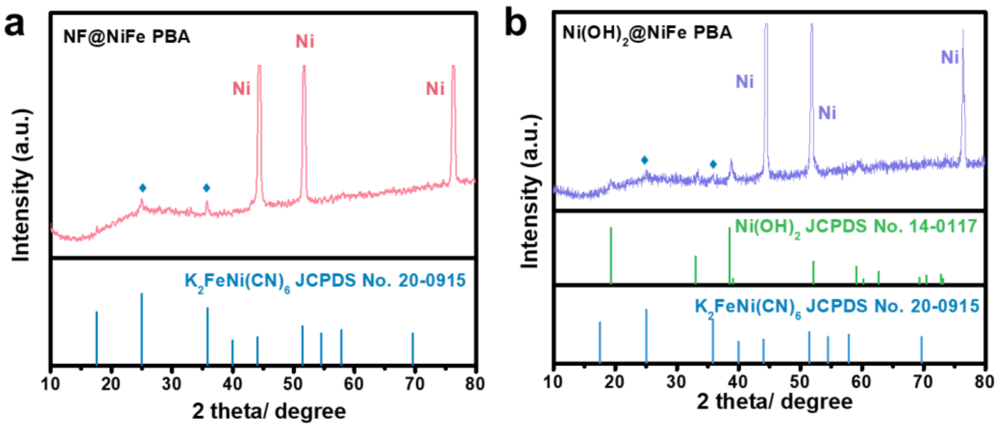

Figure S1. XRD patterns of NF@NiFe PBA and $Ni(OH)_2$@NiFe PBA.

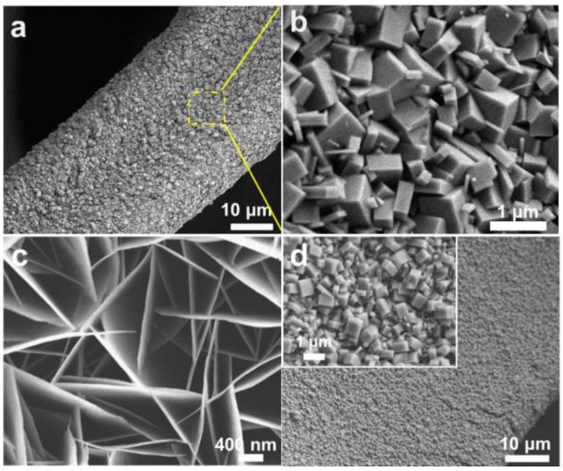

Figure S2. FESEM images of (a, b) NF@NiFe PBA, (c) $Ni(OH)_2$, and (d) $Ni(OH)_2$@NiFe PBA at different magnifications.

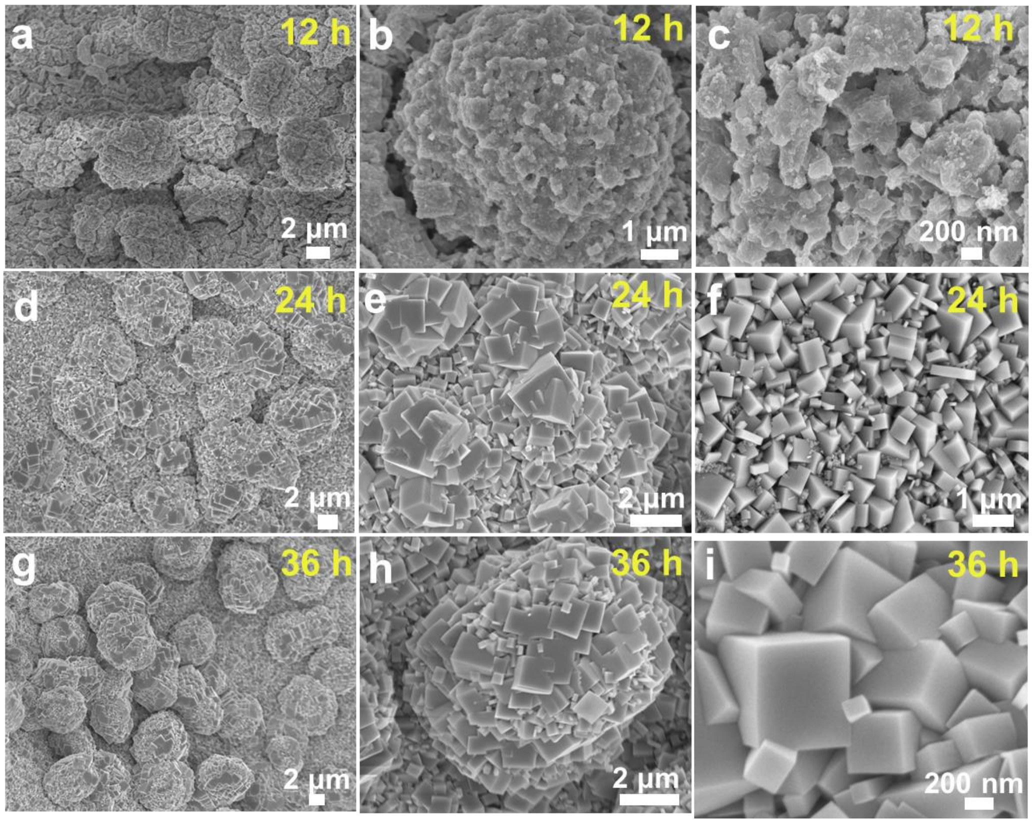

Figure S3. FESEM images of Ni_3S_2@NiFe PBA with different $K_3[Fe(CN)_6]$ etching times.

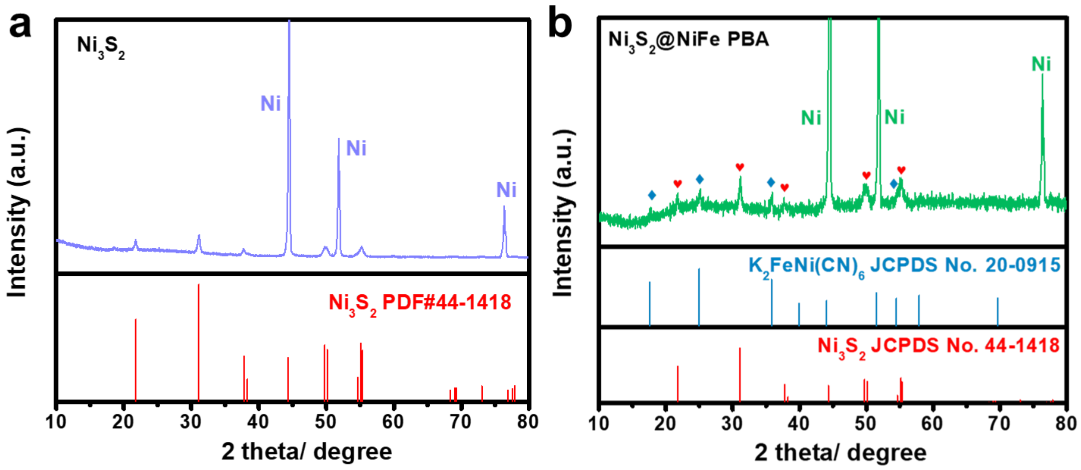

Figure S4. XRD patterns of (a) Ni_3S_2 and (b) Ni_3S_2@NiFe PBA.

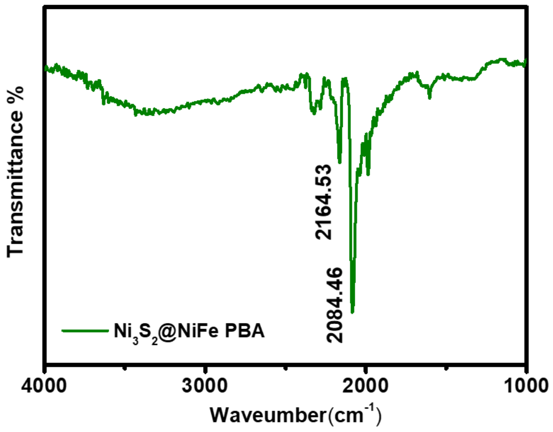

Figure S5. FT-IR spectra of Ni_3S_2@NiFe PBA.

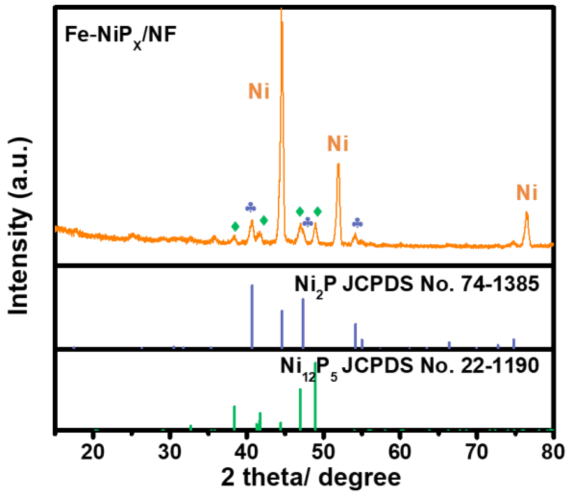

Figure S6. XRD patterns of Fe-NiP_x/NF.

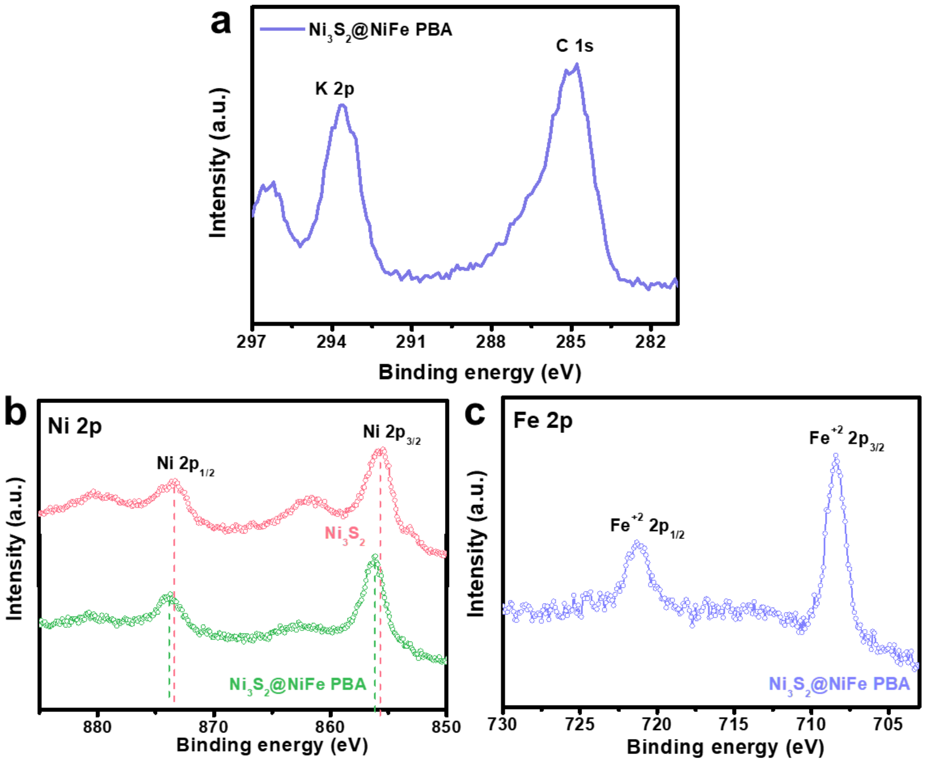

Figure S7. The high-resolution XPS of (a) C 1s, (b) Ni 2p, and (c) Fe 2p for Ni_3S_2@NiFe PBA.

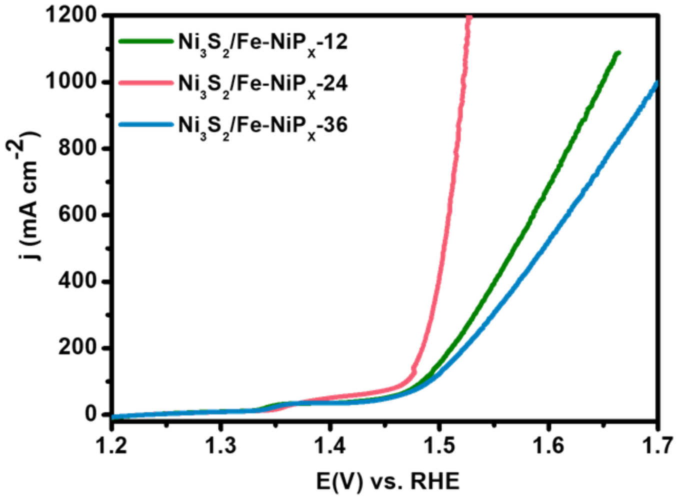

Figure S8. (a) OER Polarization curves of Ni_3S_2/Fe-NiP_x/NF with different etch time (12, 24, 36 h) by $K_3[Fe(CN)_6]$

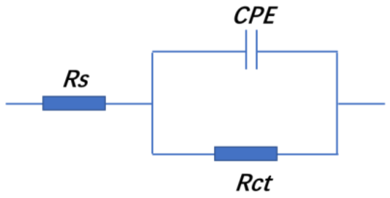

Figure S9. The equivalent circuit model for EIS Nyquist plots.

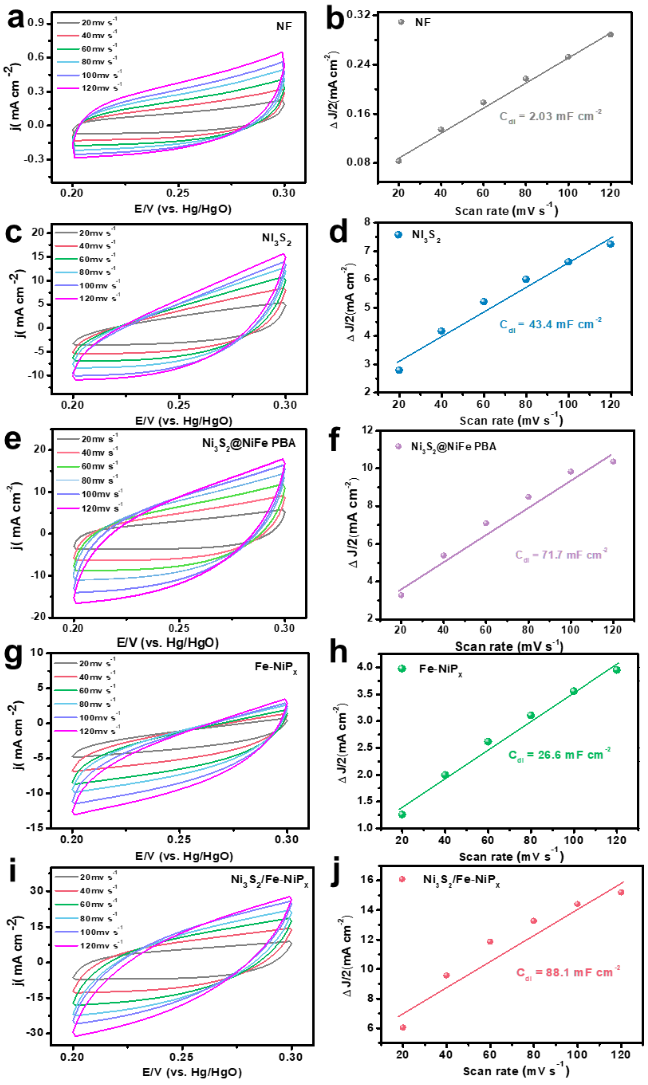

Figure S10. CV curves in the region of 0.2 - 0.3 V versus Hg/HgO at scan rates ranging from 20 mVs^{-1} to 120 mV s^{-1} and Corresponding calculated C_{dl} values for (a,b) NF, (c,d) Ni_3S_2, (e,f) Ni_3S_2@NiFe PBA, (g,h) Fe-NiP_x/NF, and (i,j) Ni_3S_2/Fe-NiP_x/NF.

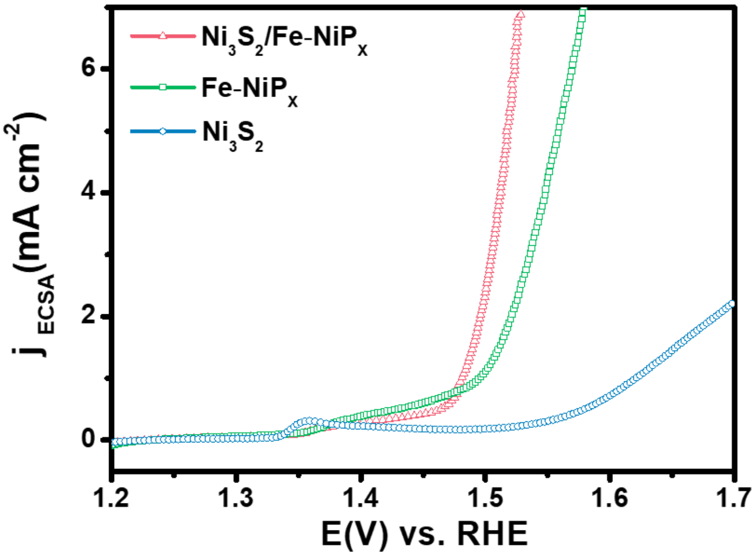

Figure S11. ECSA-normalized LSV curves for OER.

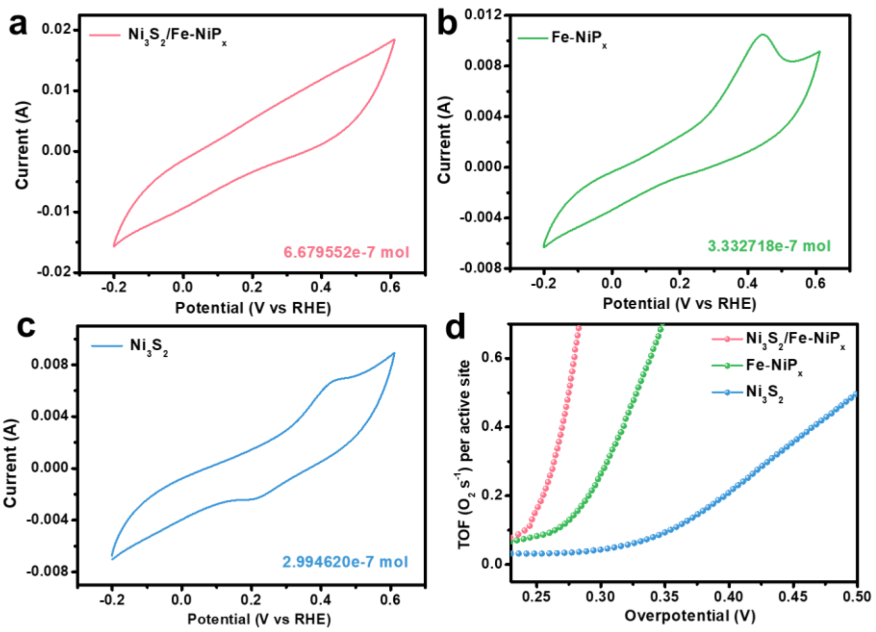

Figure S12. Cyclic voltammogram (CV) curves of (a) Ni_3S_2/Fe-NiP_x/NF, (b) Fe-NiP_x/NF, (c) Ni_3S_2/NF in 1M PBS (PH=7) with a scan rate of 50 mV s^{-1}, (d) calculated O_2 TOF values.

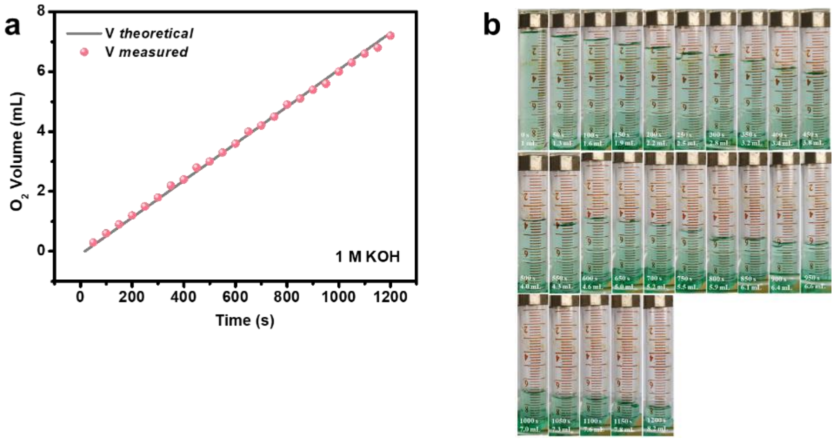

Figure S13. (a) Measured and calculated volume of O_2 for Ni_3S_2/Fe-NiP_x/NF in 1M KOH. (b) Digital photographs of collected O_2 in 1M KOH.

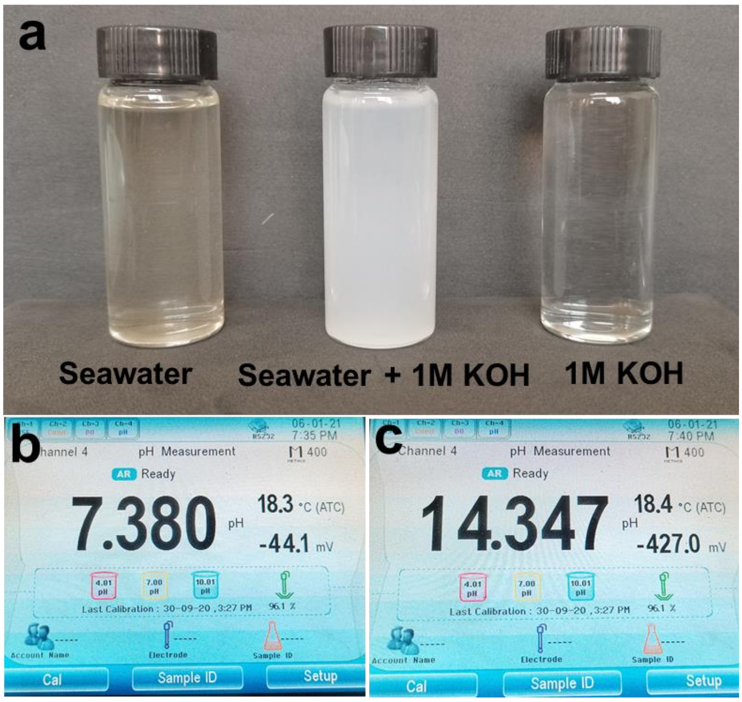

Figure S14. (a) Picture of natural seawater, alkaline natural seawater (seawater + 1M KOH) and 1M KOH solution. PH value of (b) seawater and (c) alkaline natural seawater.

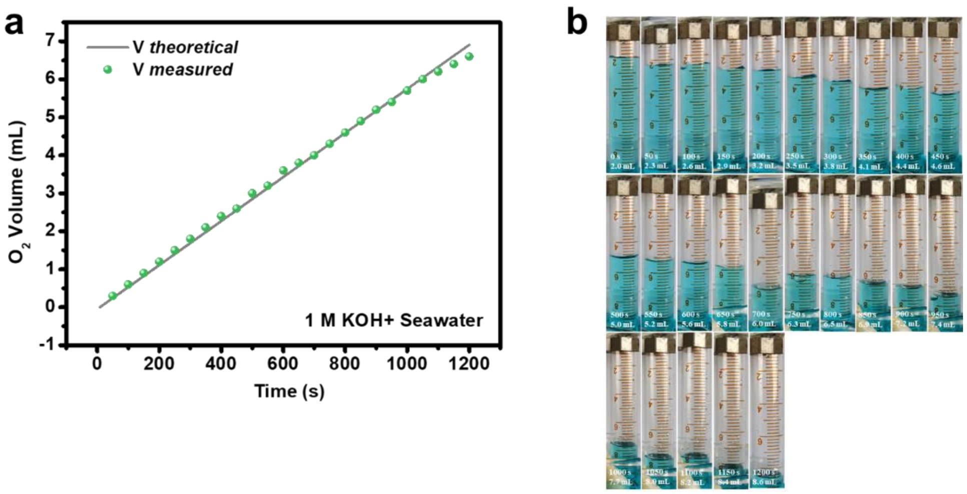

Figure S15. (a) Measured and calculated volume of O_2 for Ni_3S_2/Fe-NiP_x/NF in 1M KOH + Seawater. (b) Digital photographs of collected O_2 in 1M KOH+ Seawater.

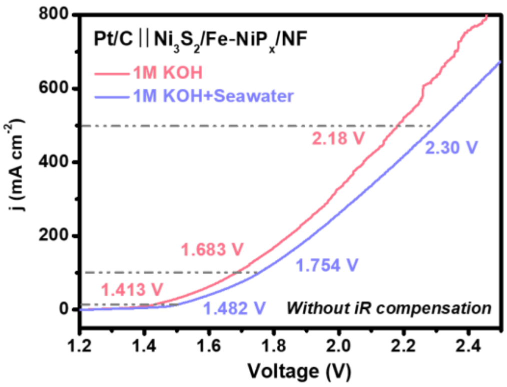

Figure S16. Polarization curves of Pt/C/NF||Ni_3S_2/Fe-NiP_x/NF for overall water/seawater splitting without iR compensation.

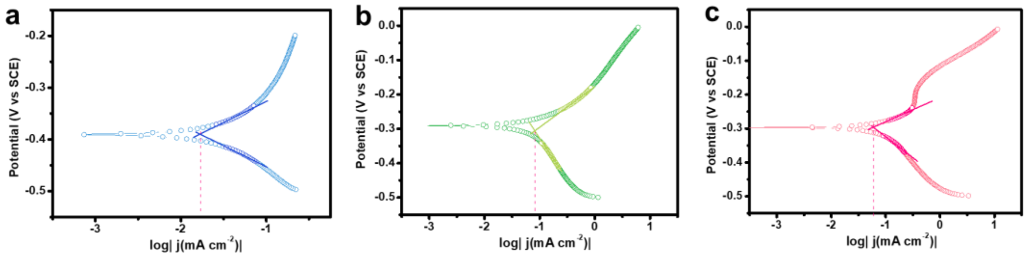

Figure S17. Corrosion polarization curves of the (a) Ni_3S_2, (b) Fe-NiP_x, and (c) Ni_3S_2/Fe-NiP_x catalysts in natural seawater.

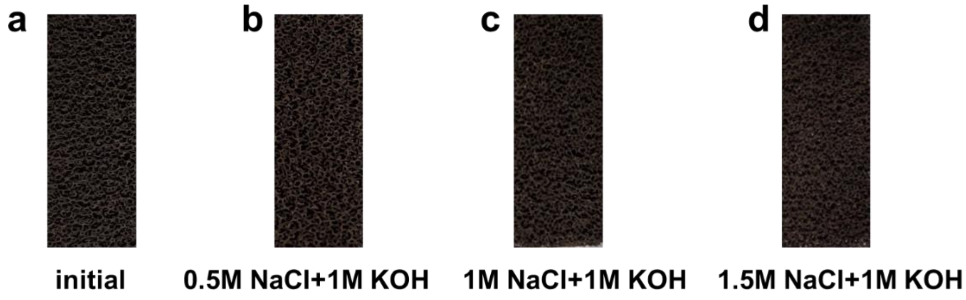

Figure S18. Optical images of Ni_3S_2/Fe-NiP_x catalysts before and after immersion in NaCl alkaline electrolyte.

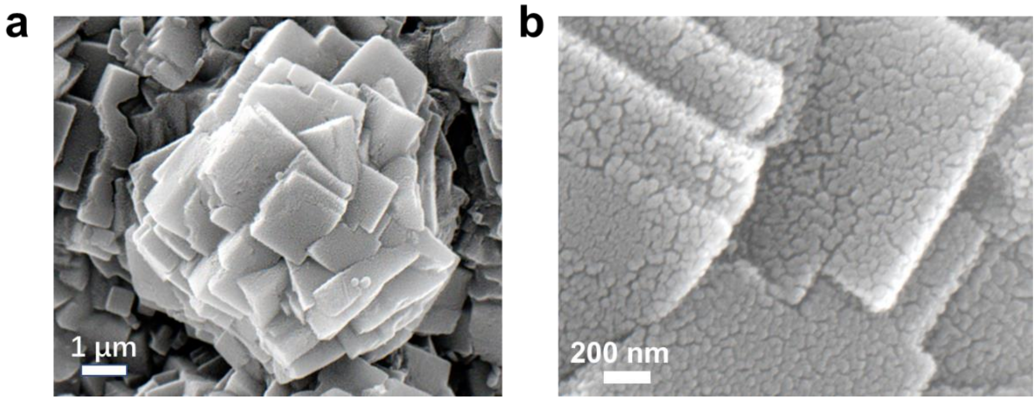

Figure S19. SEM images of Ni_3S_2/Fe-NiP_x catalysts after immersion in 1.5 M NaCl alkaline electrolyte for 20 days.

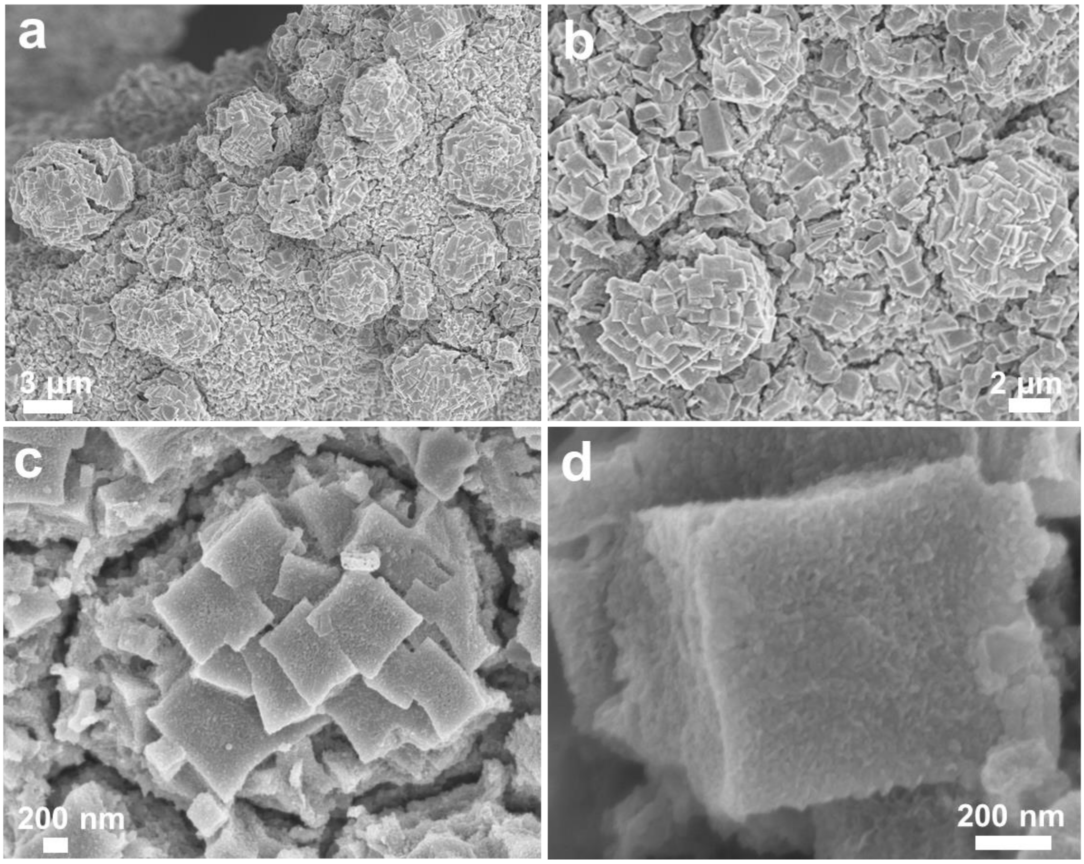

Figure S20. (a) FESEM image of Ni_3S_2/Fe-NiP_x/NF after OER test in 1M KOH solution, and (b-d) alkaline natural seawater at different magnifications.

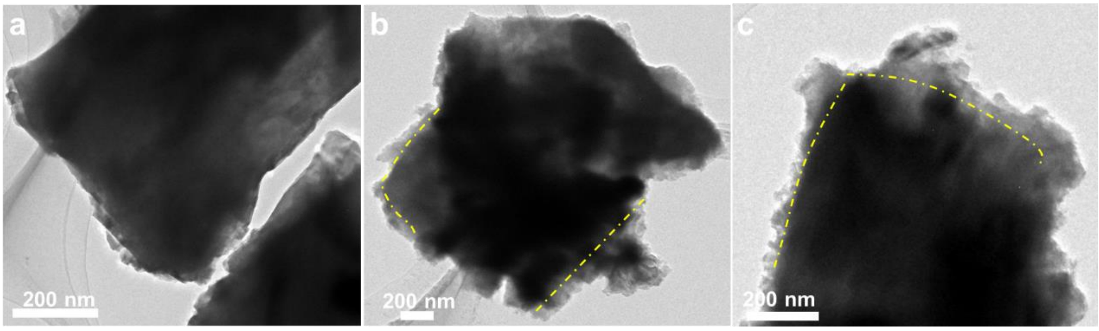

Figure S21. (a) TEM images of Ni_3S_2/Fe-NiP_x/NF, (b) Ni_3S_2/Fe-NiP_x/NF after OER test in 1M KOH solution, and (c) Ni_3S_2/Fe-NiP_x/NF after OER test in alkaline natural seawater.

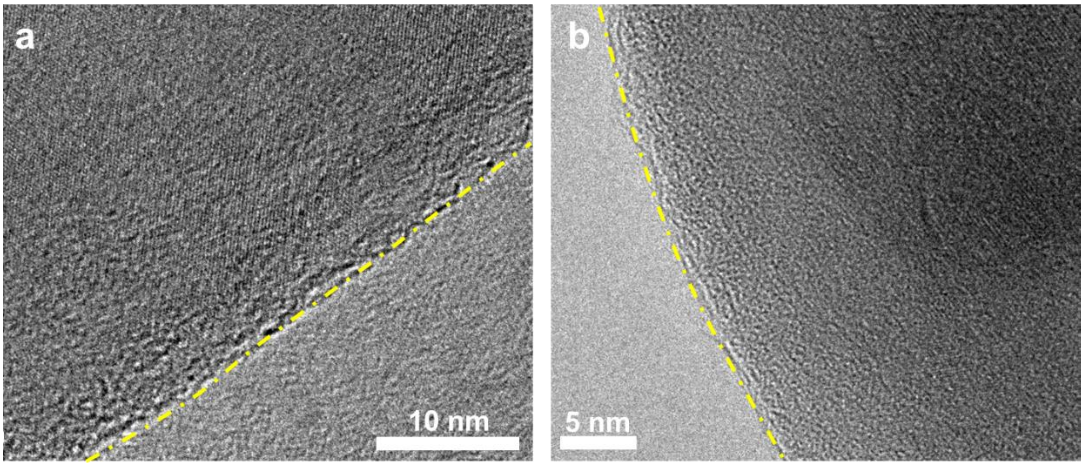

Figure S22. High-resolution TEM images of Ni_3S_2/Fe-NiP_x/NF.

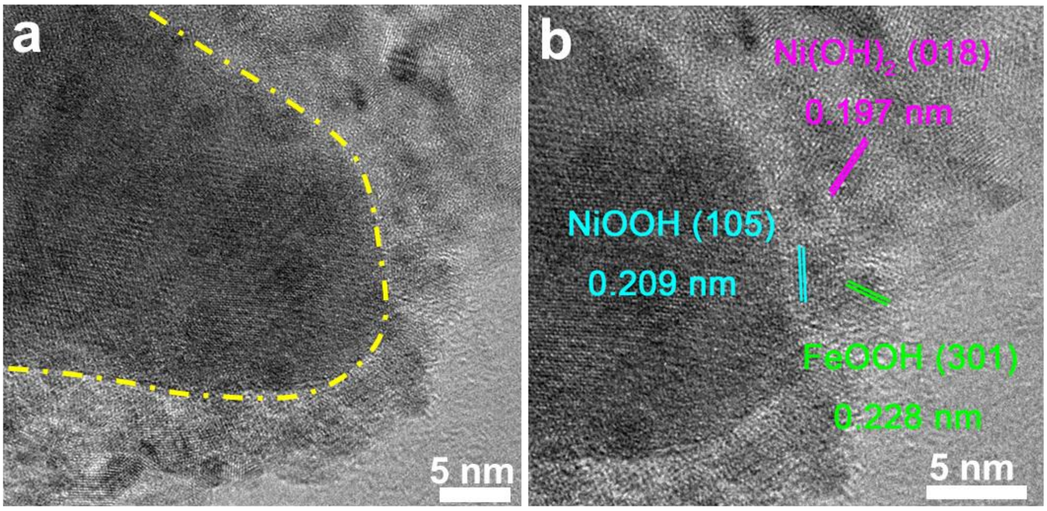

Figure S23. High-resolution TEM images of Ni_3S_2/Fe-NiP_x/NF after OER test in alkaline natural seawater.

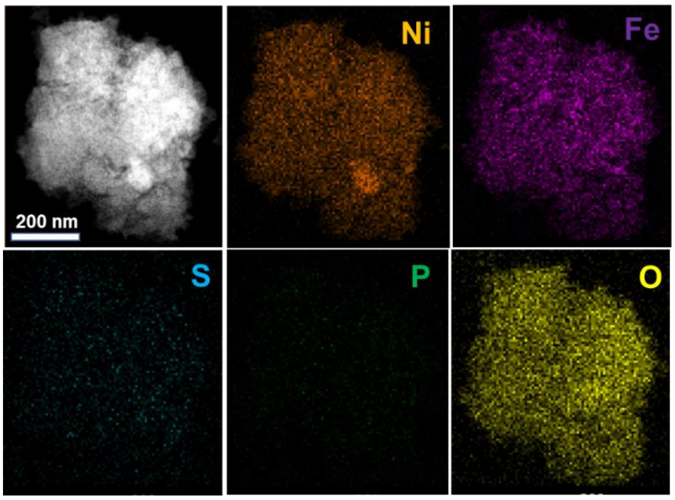

Figure S24. STEM image and the corresponding element mappings of Ni_3S_2/Fe-NiP_x/NF after OER test in 1M KOH solution.

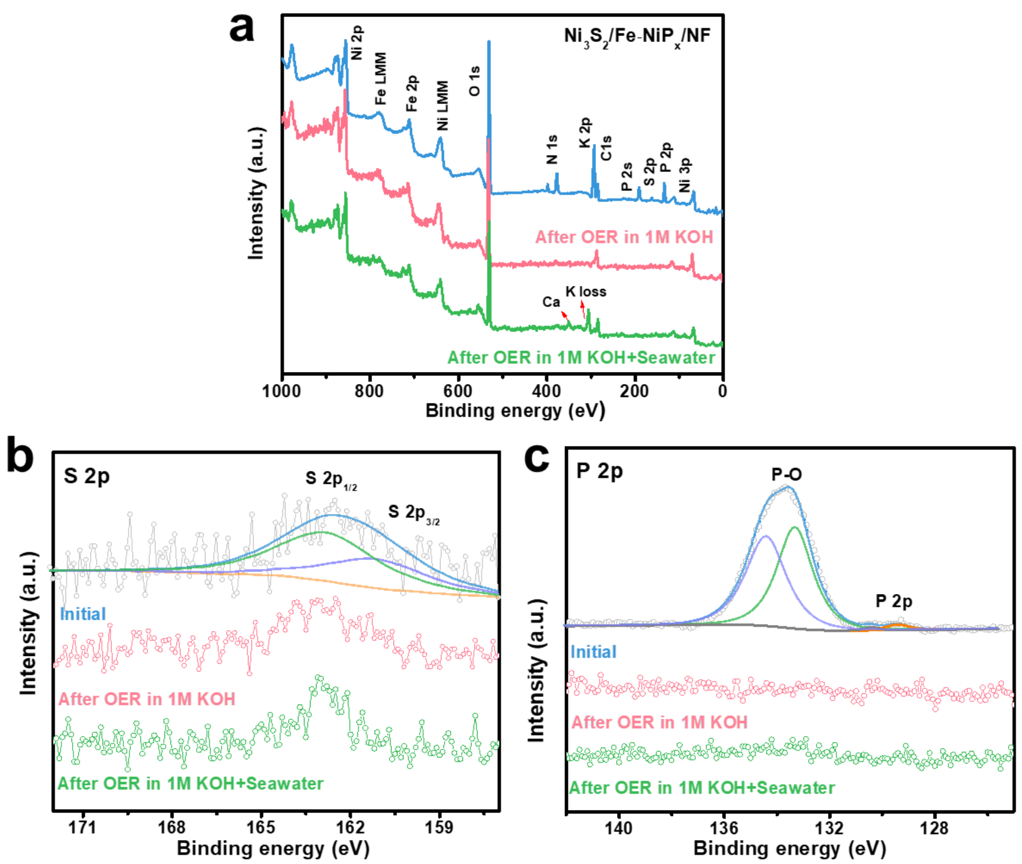

Figure S25. (a) XPS survey pattern and the high-resolution XPS of (b) S 2p, and (c) P 2p of Ni_3S_2/Fe-NiP_x/NF before and after OER test.

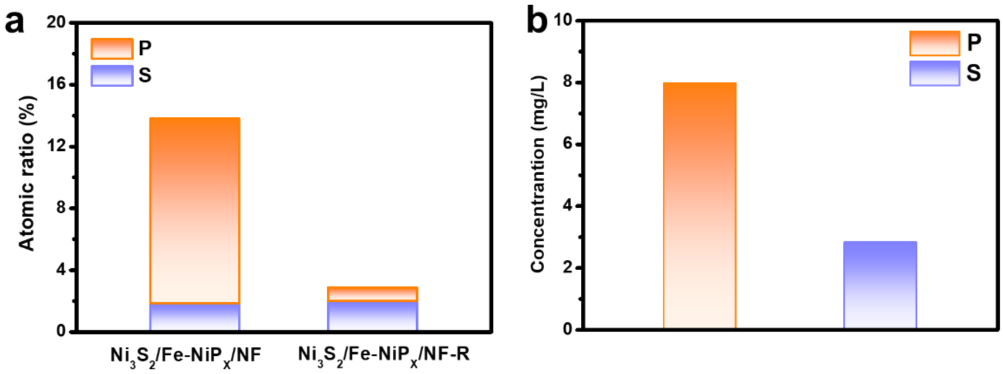

Figure S26. (a) Atomic ratio variation of Ni_3S_2/Fe-NiP_x/NF before and after reconstruction measured by XPS. (b) ICP-OES characterization of the electrolyte for Ni_3S_2/Fe-NiP_x/NF after OER stability test.

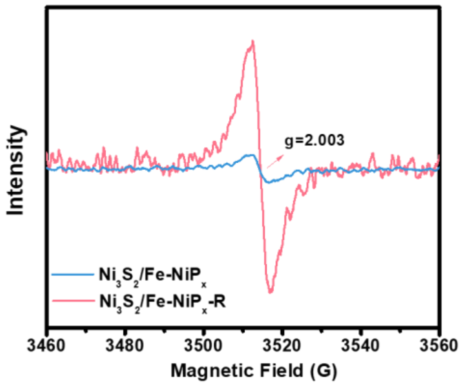

Figure S27. ESR spectra of Ni_3S_2/Fe-NiP_x/NF before and after OER (Ni_3S_2/Fe-NiP_x-R).

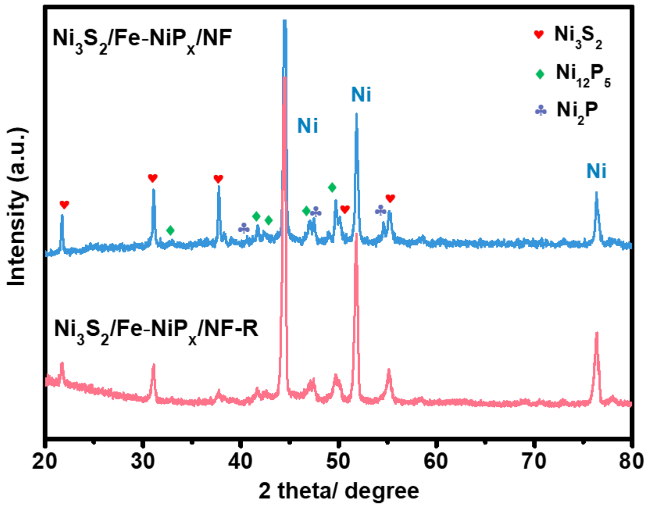

Figure S28. XRD patterns of Ni_3S_2/Fe-NiP_x/NF before and after OER test in 1M KOH.

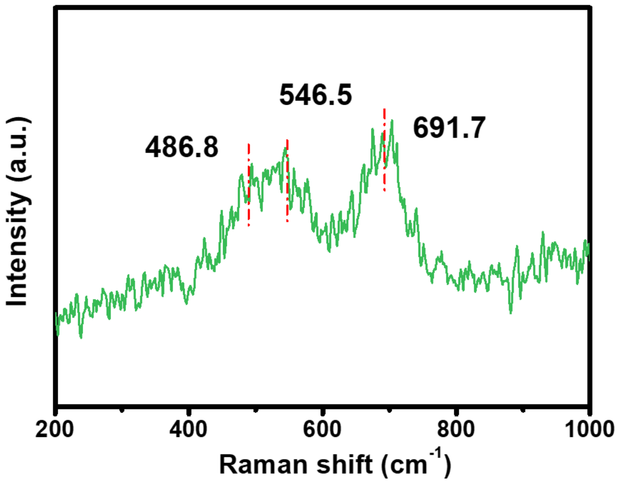

Figure S29. Raman spectra of Ni_3S_2/Fe-NiP_x/NF after OER test in 1M KOH.

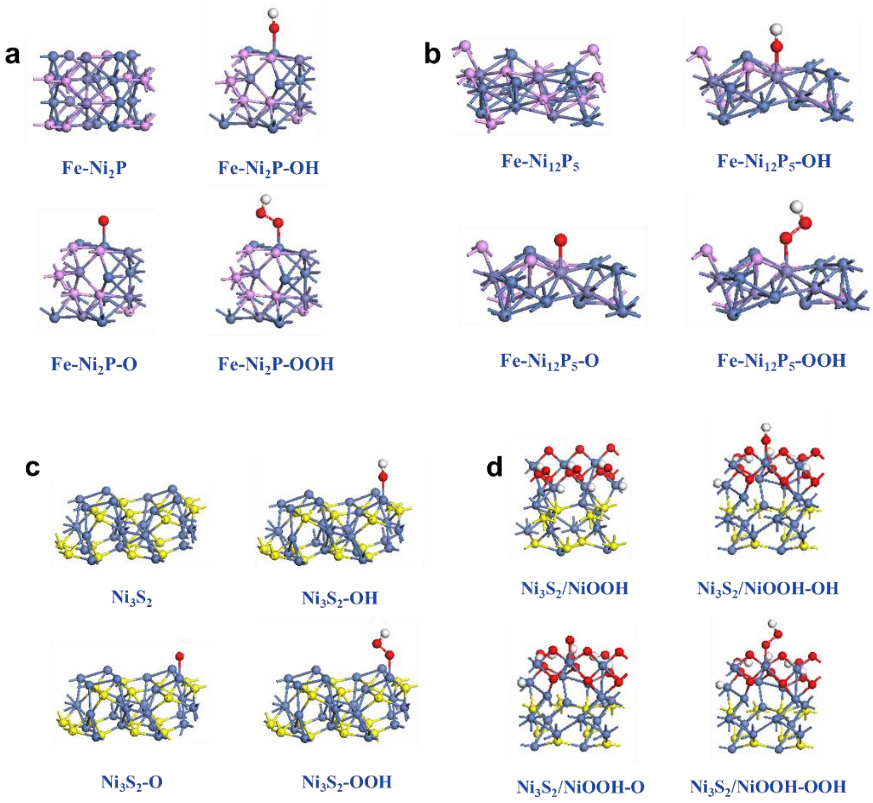

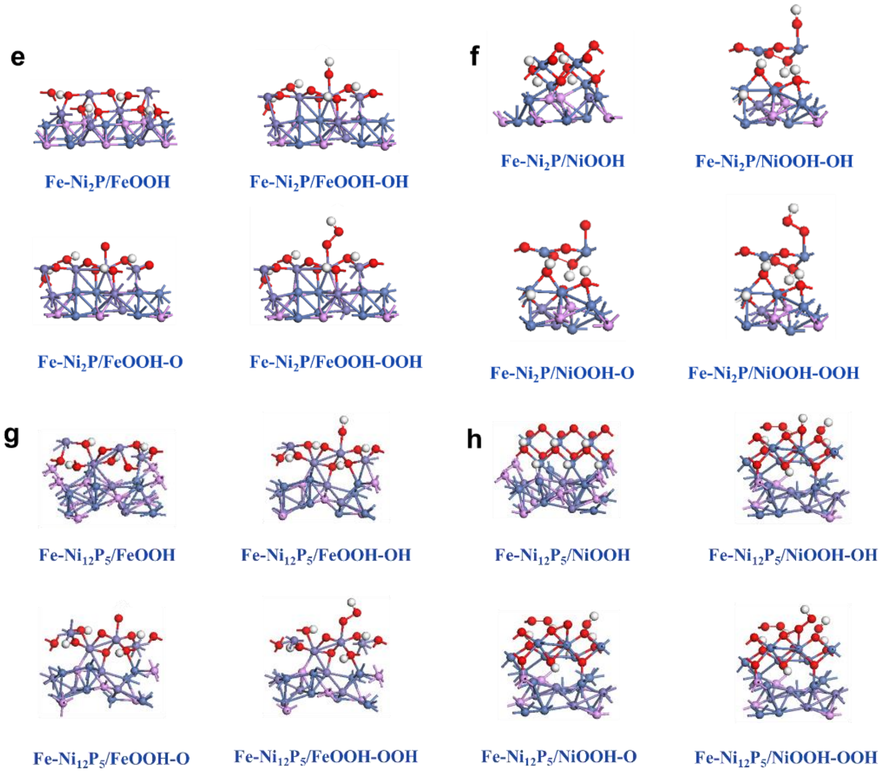

Figure 30. Theoretical structure models of OH, O, and OOH intermediates adsorbed on the (a) Fe-Ni_2P; (b) Fe-$Ni_{12}P_5$; (c) Ni_3S_2; (d) Ni_3S_2/NiOOH; (e) Fe-Ni_2P/FeOOH; (f) Fe-Ni_2P/NiOOH; (g) Fe- $Ni_{12}P_5$/FeOOH; (h) Fe- $Ni_{12}P_5$/NiOOH.

Supplementary Tables

Table S1. Comparison of OER activity of Ni_3S_2/Fe-NiP_x/NF in 1M KOH and other alkaline simulated and natural seawater with other advanced reported non-noble metal electrocatalysts.

Catalysts	Support/ working area (cm^2)	Electrolyte	Overpotential @j (mV @ mA cm^{-2})	Ref.

**Ni_3S_2/Fe-NiP_x/NF**	NF/0.25	1M KOH<br>1M KOH + seawater<br>1M KOH + 0.5M NaCl	270@500<br>291@1000<br>336@500<br>351@1000<br>285@500<br>310@1000	This work
NiMoN@NiFeN	NF/0.3-0.45	1M KOH<br>1M KOH + seawater<br>1M KOH + 0.5M NaCl	277@100<br>337@500<br>369@500<br>398@1000<br>347@500<br>377@1000	[7]
S-(Ni,Fe)OOH	NF/~	1M KOH<br>1M KOH + seawater<br>1M KOH + 0.5M NaCl	281@100<br>398@500<br>462@1000<br>339@500<br>378@1000	[8]
Ni_2P-Fe_2P/NF	NF/~	1M KOH<br>1M KOH + seawater	261@100<br>337@1000<br>305@100<br>431@1000	[9]
Fe,P-$NiSe_2$	NF/0.785	1M KOH	266@100<br>317@500	[10]
B-Co_2Fe LDH	NF/~	1M KOH<br>1M KOH + seawater	289@500<br>309@1000<br>376@500<br>415@1000	[11]
NiFe/NiS_x-Ni	NF/1	1M KOH + 0.5M	400@300	[12]

		NaCl	1500@380	
S,P-(Ni,Mo,Fe)OOH/ NiMoP/wood aerogel	wood aerogel/~	1M KOH<br>1M KOH + seawater<br>1M KOH + 0.5M NaCl	279@100<br>262@100<br>297@500<br>286@100<br>320@500	[13]
NiCoS	NF/~	1M KOH<br>1M KOH + seawater<br>1M KOH + 0.5M NaCl	270@100<br>360@100<br>440@500<br>360@500<br>430@1000	[14]
CoP_x@FeOOH	NF/0.5-0.8	1M KOH<br>1M KOH + seawater	254@100<br>292@500<br>303@800<br>283@100<br>337@500<br>354@800	[15]
(Fe-Ni)Co_x-OH/Ni_3S_2	NF/1	1M KOH	280@100	[16]
$Fe(PO_3)_2$	NF/~	1M KOH	265@500<br>300@1705	[17]
Se-doped FeOOH	IF/~	1M KOH	279@100<br>348@500	[18]
Cu@NiFe LDH	CF/~	1M KOH	281@100<br>311@500	[19]
a-NiFe-OH/NiFeP/NF	NF/0.75	1M KOH	199@10<br>258@300	[20]

*Note: nickel foam-NF; iorn foam-IF; copper foam-CF; CC: carbon cloth. “~” symbol

indicates lack of relevant information in the citation

References

[1] G. Zhao, K. Rui, S. X. Dou, W. Sun, *Adv. Funct. Mater.* **2018**, *28*, 1803291.

[2] G. Kresse, J. Hafner, *Phys. Rev. B* **1993**, *47*, 558.

[3] G. Kresse, J. Hafner, *Phys. Rev. B* **1994**, *49*, 14251.

[4] J. P. Perdew, K. Burke, M. Ernzerhof, *Phys. Rev. Lett.* **1996**, *77*, 3865.

[5] Y. Yao, S. Hu, W. Chen, Z.-Q. Huang, W. Wei, T. Yao, R. Liu, K. Zang, X. Wang, G. Wu, W. Yuan, T. Yuan, B. Zhu, W. Liu, Z. Li, D. He, Z. Xue, Y. Wang, X. Zheng, J. Dong, C.-R. Chang, Y. Chen, X. Hong, J. Luo, S. Wei, W.-X. Li, P. Strasser, Y. Wu, Y. Li, *Nat. Catal.* **2019**, *2*, 304.

[6] C. Ling, L. Shi, Y. Ouyang, X. C. Zeng, J. Wang, *Nano Lett.* **2017**, *17*, 5133.

[7] L. Yu, Q. Zhu, S. Song, B. McElhenny, D. Wang, C. Wu, Z. Qin, J. Bao, Y. Yu, S. Chen, Z. Ren, *Nat Commun* **2019**, *10*, 5106.

[8] L. Yu, L. Wu, B. McElhenny, S. Song, D. Luo, F. Zhang, Y. Yu, S. Chen, Z. Ren, *Energy Environ. Sci.* **2020**, *13*, 3439.

[9] L. Wu, L. Yu, F. Zhang, B. McElhenny, D. Luo, A. Karim, S. Chen, Z. Ren, *Adv. Funct. Mater.* **2020**, *31*.

[10] J. Chang, G. Wang, Z. Yang, B. Li, Q. Wang, R. Kuliiev, N. Orlovskaya, M. Gu, Y. Du, G. Wang, Y. Yang, *Adv. Mater.* **2021**, *33*, e2101425.

[11] L. Wu, L. Yu, Q. Zhu, B. McElhenny, F. Zhang, C. Wu, X. Xing, J. Bao, S. Chen, Z. Ren, *Nano Energy* **2021**, *83*.

[12] Y. Kuang, M. J. Kenney, Y. Meng, W. H. Hung, Y. Liu, J. E. Huang, R. Prasanna, P. Li, Y. Li, L. Wang, M. C. Lin, M. D. McGehee, X. Sun, H. Dai, *Proc Natl Acad Sci U S A* **2019**, *116*, 6624.

[13] H. Chen, Y. Zou, J. Li, K. Zhang, Y. Xia, B. Hui, D. Yang, *Appl. Catal. B Environ.* **2021**, *293*.

[14] C. Wang, M. Zhu, Z. Cao, P. Zhu, Y. Cao, X. Xu, C. Xu, Z. Yin, *Appl. Catal. B Environ.* **2021**, *291*.

[15] L. Wu, L. Yu, B. McElhenny, X. Xing, D. Luo, F. Zhang, J. Bao, S. Chen, Z. Ren, *Appl. Catal. B Environ.* **2021**, *294*.

[16] Q. Che, Q. Li, X. Chen, Y. Tan, X. Xu, *Appl. Catal. B Environ.* **2020**, *263*.

[17] H. Zhou, F. Yu, J. Sun, R. He, S. Chen, C. W. Chu, Z. Ren, *Proc Natl Acad Sci U S A* **2017**, *114*, 5607.

[18] S. Niu, W. J. Jiang, Z. Wei, T. Tang, J. Ma, J. S. Hu, L. J. Wan, *J Am Chem Soc* **2019**, *141*, 7005.

[19] L. Yu, H. Zhou, J. Sun, F. Qin, F. Yu, J. Bao, Y. Yu, S. Chen, Z. Ren, *Energy Environ. Sci.* **2017**, *10*, 1820.

[20] H. Liang, A. N. Gandi, C. Xia, M. N. Hedhili, D. H. Anjum, U. Schwingenschlögl, H. N. Alshareef, *ACS Energy Lett.* **2017**, *2*, 1035.
